# An Overview of Current Knowledge on the Properties, Synthesis and Applications of Quaternary Chitosan Derivatives

**DOI:** 10.3390/polym12122878

**Published:** 2020-11-30

**Authors:** Emanuelle Dantas Freitas, Celso Fidelis Moura Jr., Jonas Kerwald, Marisa Masumi Beppu

**Affiliations:** Department of Materials and Bioprocess Engineering, School of Chemical Engineering, University of Campinas, Campinas, São Paulo 13083-852, Brazil; manuddf@gmail.com (E.D.F.); fideliscelso@gmail.com (C.F.M.J.); jkerwald@ucs.br (J.K.)

**Keywords:** quaternized chitosan, chitosan derivatives, quaternization, TMC, HTCC, pyridinium salts, phosphonium salts

## Abstract

Chitosan, a chitin-derivative polysaccharide, known for its non-toxicity, biocompatibility and biodegradability, presents limited applications due to its low solubility in neutral or basic pH medium. Quaternization stands out as an alternative to modify this natural polymer, aiming to improve its solubility over a wide pH range and, consequently, expand its range of applications. Quaternization occurs by introducing a quaternary ammonium moiety onto or outside the chitosan backbone, via chemical reactions with primary amino and hydroxyl groups, under vast experimental conditions. The oldest and most common forms of quaternized chitosan involve N,N,N-trimethyl chitosan (TMC) and N-[(2-hydroxy-3-trimethyl ammonium) propyl] chitosan (HTCC) and, more recently, quaternized chitosan by insertion of pyridinium or phosphonium salts. By modifying chitosan through the insertion of a quaternary moiety, permanent cationic charges on the polysaccharide backbone are achieved and properties such as water solubility, antimicrobial activity, mucoadhesiveness and permeability are significantly improved, enabling the application mainly in the biomedical and pharmaceutical areas. In this review, the main quaternized chitosan compounds are addressed in terms of their structure, properties, synthesis routes and applications. In addition, other less explored compounds are also presented, involving the main findings and future prospects regarding the field of quaternized chitosans.

## 1. Introduction

Chitosan is a polycationic polymer (pKa = 6.2–6.8) obtained by partial chitin N-deacetylation under alkaline conditions, thus consisting of a linear mucopolysaccharide formed by β-(1 → 4)-2-amino-2-deoxy-d-glucose and β-(1 → 4)-2-acetamide-2-deoxy-d-glucose copolymers. This cationic character is the basis of most of its applications as an antimicrobial compound in several fields, including food, agriculture, biomedicine and textiles [1]. As a result that it presents itself as a biodegradable [2] and biocompatible [3] material, chitosan has been arousing the interest of several research groups due to its physicochemical properties and unique bioactivity [4,5,6,7]. However, chitosan has low solubility in aqueous solutions and organic solvents, in addition to having a short useful life, thus limiting its applicability [8].

One of the methodologies used to solve this limitation is the quaternization of chitosan. Chitosan quaternization consists of replacing the chitosan amino terminals with quaternary terminals or by inserting functional cationic groups [9,10]. This quaternization improves the solubility of chitosan, in addition to enhancing its antimicrobial action [11]. The introduction of permanent positive charges in the chitosan chain can be achieved by preparing a quaternary ammonium chitosan salt, covalently adding a substituent containing a quaternary ammonium group or quaternizing the amino group of the original polymer [12]. This permanent introduction of positive charges in the polymer, regardless of the pH of the aqueous medium, provides solubility in water as well as better antimicrobial activity [13,14]. This improvement in antimicrobial activity may be related to the fact that the positive charges in chitosan are captured by cells, inhibiting the transition of DNA and the synthesis of RNA and proteins, or even, the disorganization and denaturation of the proteins of the microorganism’s membrane caused by the interaction between positive chitosan groups and the negative charges of the microorganism’s membrane and therefore causing cell death [15,16,17].

The most studied and simple form of quaternary ammonium chitosan derivative is N,N,N-trimethyl chitosan (TMC) [18]. This derivative is obtained by reaction with methyl iodide, sodium iodide or sodium hydroxide [19]. The insertion of portions of quaternary ammonium outside the chitosan structure by an alkylation reaction is the second most common way to quaternize chitosan, forming the compound N-[(2-hydroxy-3-trimethyl ammonium) propyl] chitosan (HTCC) and it is commonly synthesized from a reaction between chitosan and gycidyltrimethyl ammonium chloride [20,21,22]. Recently, new quaternized chitosan derivatives with pyridinium salts and phosphonium salts have been gaining interest from several research groups due to the antimicrobial properties presented by these quaternary salts in addition to providing better solubility to chitosan [23,24,25,26,27]. These derivatives, based on the literature, have been shown to be quite efficient in microbial activities when compared to chitosan [28,29,30,31,32], in addition to being able to be applied in areas such as cosmetic agents [33], pharmaceuticals and biomedical [34,35,36,37], food industry [38,39], antimicrobial agent [24,40,41] and wastewater treatment [42,43].

This review focuses on presenting the main quaternized compounds of chitosan, from the most used, such as TMC and HTCC, to those that have aroused a greater interest of researchers, such as the quaternized chitosan derivatives with pyridinium or phosphonium salts. We addressed them in terms of their structure, properties, synthesis routes and applications. In addition, other less explored compounds are also presented, involving the main findings and future perspectives in the field of quaternized chitosans.

## 2. TMC

### 2.1. Structure and Properties of TMC

**Figure 1:**
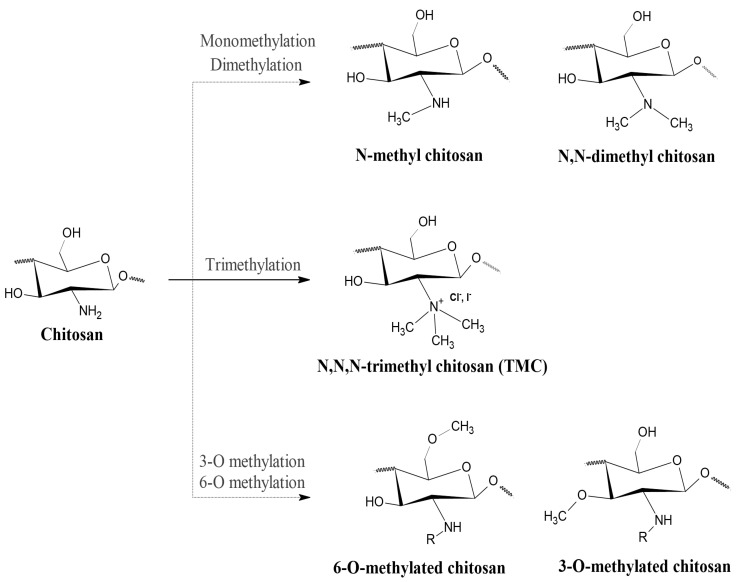
Chitosan, N,N,N-trimethyl chitosan (TMC) and its N-methylated and N,O-methylated derivatives.

N,N,N-trimethyl chitosan (TMC) is an N-alkyl derivative of chitosan, which is obtained through the quaternization (methylation) of amino group in C-2 position of the polymer’s backbone. During this process, other similar compounds are frequently produced, such as 6-O methylated and 3-O methylated TMC, and N-methylated and N,N-dimethylated chitosan [44], shown in Figure 1. These subproducts are seldom, if ever, desired and their presence in the final product may result in unhoped-for properties. Among the quaternary chitosan compounds, TMC is the most studied one, mainly because of the simplicity of its synthesis methods [18].

TMC was first synthesized by Terayama, H. and Terayama, E. [45] and it was called Macramin. Its antibacterial activity was first reported by Hatta et al. [46]. Much later experiments carried out by Muzzarelli and Tanfani [47] were able to produce TMC through different steps, but the compound displayed low water solubility. A year later, Domard et al. [48] developed a slightly different synthesis method that was able to obtain a water-soluble compound, independently of the pH. Several studies were based on these ones and attempted to improve these preparation approaches. The highlights and results obtained will be described with more detail in the next subsection.

As a chitosan derivative, TMC preserves a lot of its original properties. However, some features can be highly improved or drastically changed with its quaternization. Differently from chitosan, TMC is soluble in neutral and alkaline media. Its solubility is influenced by several factors. First, the deacetylation degree (DD) of the original chitosan impacts directly in the quantity of amino groups available for modification, which also has a direct influence on the degree of quaternization (DQ). The DQ is also an important factor on the solubility of TMC. The higher the DQ is, the higher is the number of substituted amino groups, meaning that the overall cationic charge will be increased and so will the solubility of the polymer [49].

The DQ of TMC can be improved by modifications on the synthesis process or repeating the methylation process multiple times. The drawback of this method is that it leads to an increase in the number of O-methylated TMC that is produced. Studies have shown that a high degree of O-methylation weakens the solubility of TMC [50,51]. In addition, prolonged synthesis process results in the production of N,N-dimethyl chitosan, which is insoluble and will hence diminish the overall solubility of TMC. This way, a higher DQ does not always result in higher solubility [52]. Thus, all of these factors must be considered during the preparation of chitosan quaternary derivatives.

The mucoadhesion potential of TMC is also improved when compared to that of chitosan [53]. This property, too, is affected by TMC’s DQ, even though the findings, so far, have been contradictory. Sandri et al. [54] reported an increase in bucal mucosal membrane adhesiveness when the material’s DQ was increased. This result may be attributed to the increase in the polymer’s overall cationic charge, which is augmented with the presence of quaternary groups. Thus, the electrostatic interaction of TMC with the negatively charged sialic acid residues present mucin, a glycoprotein that composes mucus, will be stronger. On the other hand, Snyman et al. [55] found a decrease in mucoadhesivity with an increase in the degree of quaternization. The authors related this to conformational changes in the structure of TMC. In a similar context, Jintapattanakit et al. [56] attributed a combination of positive charge density, steric hindrance of pendent groups and molecular weight to be highly influential on mucoadhesive properties of the polymer.

Interactions of TMC with the epithelial membrane are also responsible for its permeation enhancing properties. The polymer is reported to aid penetration of hydrophilic and/or high-molecular-weight molecules across the intestinal epithelium, especially peptides and proteins. This is associated with the opening of the intercellular tight junctions [57]. The influence of DQ in the transport of [${}^{14}$C]-mannitol across intestinal Caco-2 cell monolayers was evaluated by Thanou et al. [58]. They reported higher transport enhancement as TMC charge density was increased, improving the paracellular permeability of intestinal epithelia. Similar results were obtained by Florea et al. [59], who assessed the enhancement of pulmonary delivery of octreotide by TMC. The authors describe pH, solubility and cationic charge density as important factors for the modulation of the paracellular barrier.

Regarding its wettability, whereas chitosan has a hydrophilic nature, N-methylated chitosan has an amphiphilic nature due to the hydrophilic character of its (N-(CH_3_)_3_) groups and the hydrophobic character of its (N-(CH_3_)_2_). Thus, almost pure TMC will present a high hydrophilic character which will decrease as more hydrophobic subproducts, such as N,N-dimethyl chitosan, are added to the system [16]. O-methylated derivatives, on the other hand, increase the overall hydrophilicity due to the formation of permanent positive charges on chitosan’s backbone [60].

The antibacterial activity of chitosan is believed to be due to its amino positive charge, which interacts with the negative charge on the surface of bacterial cells [61]. As a result of this, TMC normally presents a higher bactericidal potential than that of its parent chitosan, increasing as its cationic charge density is augmented with presence of quaternary groups. TMC has been demonstrated to have antibacterial activity against Gram-negative bacteria: *Escherichia coli* [19,62,63], *Enterococcus facialis, Pseudomonas aeruginosa* [62], *Salmonella enterica* [64] and Gram-positive bacteria: *Staphylococcus aureus* [62,63,65], *Listeria innocua* [66], *Bacillus subtilis* [64]. Regarding TMC’s activity against Gram-positive bacteria, Sadeghi et al. [67] reported that the TMC derivatives with the highest zeta potentials were the ones that presented the higher value of minimum inhibitory concentration (MIC). This is probably because the more positively charged the polymers are, the higher is their ability to bind to the negative peptidoglycans on the bacterial cell wall and, therefore, the probability of autolysis occurrence is increased. Alkyl chain length, molecular weight and pH have also been reported to influence the bactericidal potential of TMC and its derivatives [63,65].

Contrarily to the other properties, the thermal stability of TMC is lower than that of the original chitosan. A kinetic study conducted by De Britto et al. [68] evaluated thermal degradation of TMC and found out that the higher the DQ is, the lower is the thermal stability of the polymer. This may be due to the fact that the methyl groups incorporated on nitrogen atoms decrease the intra-chain strength, especially those from H-bonds. In addition, TMC’s hydrophilic character results in higher water content, which also weakens interchain interactions [53].

### 2.2. Preparation Methods

**Figure 2:**
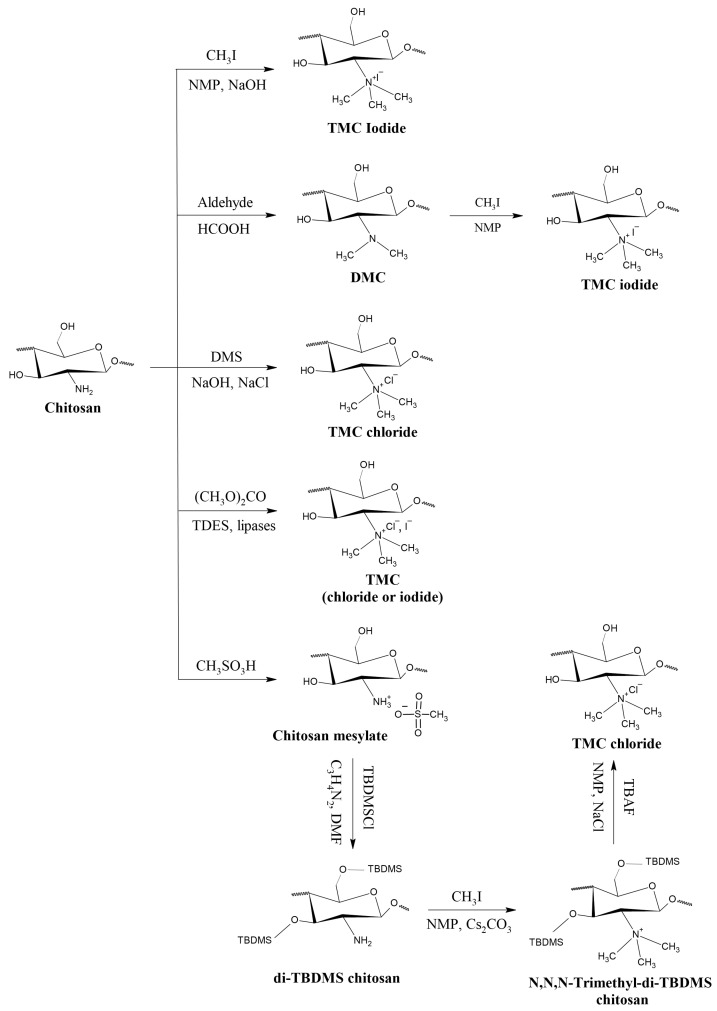
TMC preparation methods summarized.

Until 2016, there were four main methods for preparing TMC, according to Wu et al. [69] and Kulkarni et al. [70]. These methods are described below, followed by alternative methods and approaches that were able to successfully produce TMC in the last four years. A scheme with a summary of the most common methodologies employed is displayed in Figure 2.

#### 2.2.1. Conventional Methods

The simplest way of obtaining TMC is by a one-step reaction of chitosan with methyl iodide (CH${}_{3}$I) under strong alkaline conditions using N-methyl-2-pyrrolidone (NMP) as a solvent and sodium iodide (NaI) as a catalyst [48]. Curti et al. [12] verified that an excess of NaOH resulted in higher trimethylation, even reaction yield was lower and O-methylation was favored. As a result of this, water solubility decreased as the alkaline agent and CH${}_{3}$I excess was increased. The average degrees of quaternization obtained in this study ranged from 10% to 45%.

By using methyl iodide as methylating agent, N,N,N-trimethyl chitosan iodide is obtained. As a result of its toxicity and, therefore, unsuitability of application in several fields, it is normally converted to TMC chloride, either by reacting with NaCl [48,71] or hydrochloric acid (HCl) [52]. In this context, Zhang et al. [72] prepared four TMC salts (citrate, acetylsalicylate, ascorbate and gallate) by dissolving iodide precursor in solutions of sodium salts with the four desired couteranions. As a result, the gallate and ascorbate derivatives presented better antioxidant activity than chitosan and TMC iodide.

Another method, initially developed by Muzzarelli and Tanfani [47], involved the synthesis of N,N-dimethyl chitosan (DMC) by reacting chitosan with formaldehyde in acidic media and following addition of sodium borohydride. Only then was CH_3_I added to the system to produce TMC. The obtained compound was water insoluble, even with a DQ as high as 60%. A modification of this method was later executed by Verheul et al. [51]. In the study, instead of sodium borohydride, DMC was synthesized using a formic acid–formaldehyde mixture. Trimethylation was then carried out with an excess of methyl iodide. Through this method, the authors were able to obtain O-methyl free water soluble TMC with no chain scission. They also stated that TMCs with DQs as high as 75% could be obtained by varying reaction time.

A variation of this method was attempted by some authors [19,65]. In these approaches, a pre-alkylation of chitosan was carried out by used different aldehydes than formaldehyde in order to obtain a Schiff-base intermediate. Later on, quaternization was carried out traditionally by using CH_3_I. By previously introducing methyl groups on the nitrogen atom, the formation of quaternary ammonium salts is facilitated along with the hindering of O-methylation. However, because of the use of different and more complex aldehydes in the pre-alkylation step, other quaternized chitosan derivatives, such as N-N-Propyl-N,N-dimethyl chitosan and N-Furfuryl-N,N-dimethyl chitosan, can be obtained along with TMC [19].

In spite of the efficiency obtained by using CH_3_I as a methylation agent, it is an expensive, volatile and toxic reagent. In addition, halide ions are difficult to separate from solution. Thus, De Britto et al. [60] synthesized TMC with the use of dimethyl sulfate (DMS). Compared to methyl iodide methods, this approach results in a less expensive, less toxic and more simple method, as DMS can act both as an agent and a solvent for the reaction, avoiding the use of NMP. In addition, TMC chloride was directly obtained as NaCl is used in the medium. In this study, the calculated DQs ranged from 15.8 to 52.5% and were found to be time and temperature dependent, with high temperatures favoring O-methylation over N-methylation.

Due to the fact that methylation agents are not selective and O-methylation occurs in almost all of the methods cited above, even if chitosan’s amino groups are much more reactive than C-3 and C-6 hydroxyl groups, it is a good strategy to protect these less reactive groups through O-silylation. First, chitosan is reacted with methanesulfonic acid (CH_3_SO_3_H) to obtain chitosan metasylate, followed by the insertion of tert-butyldimethylsilyl (TBDMS) groups, producing 3,6-di-O-tert-butyldimethylsilyl-chitosan. This compound displays excellent solubility in a number of common organic solvents [73]. Once the hydroxyl groups are protected and O-methylation can no longer occur, the methylation of this chitosan derivative is executed by using CH${}_{3}$I. According to Benediktsdóttir et al. [74], such procedure leads to a product with 100% trimethylation, composed of N,N,N-Trimethyl-di-TBDMS chitosan. Finally, the hydroxyl groups can be deprotected through the use of a tetrabutylammonium fluoride (TBAF) solution in NMP. The authors also reported, for the first time, a full N,N,N-trimethyl chitosan without the presence of O-methylated or N-mono or dimethylated derivatives.

#### 2.2.2. Alternative Methods and Approaches

As a result that conventional agents used for methylation of chitosan, such as alkyl halide or dimethyl sulfate, are highly toxic and carcinogenic, Wu et al. [75] proposed a novel green approach for CHI quaternization. The authors quaternized chitosan by using dimethyl carbonate as a methylation reagent in an ionic liquid (acting as a solvent and a catalyst). Even though the preparation process is not optimized and still needs improvement, especially in order to obtain higher DQs, the authors were able to produce TMC without the occurrence of O-methylation.

Mahajan et al. [76] explored a complete green approach to the synthesis of TMC by using two different lipases, (*Burkholderia cepacia* and *Candida rugosa*), as biocatalysts and dimethyl carbonate as the green methylating agent, in a reaction medium comprising of ternary deep eutectic solvents. As a result, whereas the lipase from *B. cepacia* selectively N-methylated the chitosan polymer, the one from *C. rugusa* yielded an O-methylated product. The quaternization degrees obtained from both lipases were of about 12.5 and 15.7%, respectively. These values are still very low when compared to the ones that are obtained through conventional methods, indicating an urgency for optimization of green methods.

### 2.3. Applications of TMC and Its Derivatives

TMC has been applied to several fields in the form of nanoparticles, films, blends, emulsions, etc. Biomedicine is, by far, the field that have been the most studied for trimethyl chitosan and its derivatives. In addition, some research has been done about TMC in environmental and food applications. In this context, some reviews have been written. Kulkarni et al. [70] wrote a review paper focused on applications of TMC in the form of nanoparticles and some other specific applications have been tracked by Mourya and Inamdar [77], Wu et al. [69] and Zhao et al. [78]. Therefore, this review will focus more on recent applications. It is important to be aware that “TMC” almost always refers to TMC iodide, but it is hardly ever specified by authors in their publications and thus the term “iodide” will be not used throughout this section. Whenever another counter-anion, such as chloride, replaces iodide, it will be explicitly stated below.

Chitosan and its derivatives have been widely studied as adsorption enhancers, especially of peptide and protein drugs. This is due to the opening of tight junctions in between epithelial cells, which facilitates the paracellular diffusion across mucosal epithelia [79]. Kotzé et al. [80] demonstrated that TMC was able to increase transport of the hydrophilic compounds [${}^{14}$C]-mannitol, a fluorescein isothiocyanate-labeled dextran and the peptide drug buserelin across Caco-2 cell monolayers. Since then, several studies have reported TMC’s efficiency as a permeation enhancer. Thanou et al. [58] evaluated the effect of the degree of quaternization of trimethyl chitosan on the absorption enhancement. The authors showed that high charge density is necessary for TMC to significantly improve the paracellular permeability, which means that the transport enhancement across intestinal epithelia is improved with higher DQs. He et al. [81] reported improved transdermal permeation of testosterone by using TMC and also verified a stronger enhancement with high DQs. Trimethyl chitosan displayed penetration enhancement towards intestinal [82], nasal [83], corneal [84] and bronchial epithelia, [85] and buccal mucosa [54].

As a result of TMC’s excellent mucoadhesive and absorption-enhancing properties, it shows potential to be used, especially, in the delivery of oral drugs. It can increase protein bioavailability in gastrointestinal environments and permeability in the intestinal barrier, in addition to its low toxicity and high susceptibility to biodegradation. In the last few years, trimethyl chitosan and its derivatives have been successfully applied in systems aiming oral delivery of insulin [86,87], natural antioxidants [88], flavonols [89], anticancer drugs, such as paclitaxel [90] and gemcitabine [91], antifungal drugs [92] and antiviral drugs [28]. In addition to oral administration, researchers have developed systems composed of TMC to deliver drugs through nasal [93], pulmonary [94] and intravenous [90].

Drug delivery can also be done through ocular administration. In this sense, efforts have been made in order to develop new drug carriers that are able to increase ocular absorption and improve drug bioavailability. Asasutjarit et al. [36] developed formulations of TMC NPs loaded with diclofenac sodium (DC) for ophthalmic use. Eye irritation tests revealed that this material is safe for use. Additionally, in vivo ophthalmic absorption studies conducted with rabbits showed that bioavailability of DC is increased and the system could be used for treatment of ocular inflammation with lower frequency of administration than that of common DC eye drops. Similarly, Li et al. [95] prepared TMC-coated lipid nanoparticles of baicalein (TMC-BAI-LNPs). In vitro and in vivo studies indicated that the material had no ocular irritation and resulted in prolongation of drug retention time in tears and improvement of its ocular bioavailability.

Cationic chitosan is able to form a polyelectrolyte complex with anionic DNA and thus it is applied to gene delivery systems. Additionally, polymeric vehicles seem more convenient than other gene delivery approaches because of efficiency and safety issues. Rahmani et al. [96] investigated DNA transfection efficiency of three chitosan derivatives, one of them being a TMC derivative (thiolated trimethyl chitosan). The authors reported that the three compounds are efficient vehicles for gene delivery and that specifically for SKOV-3 and MCF-7 cells, TMC exhibited the highest transfection efficiency of DNA nanocomplexes. According to this study, the thiol group can enhance the transfection process, even though cell type is the determining factor in a delivery system. Baghaei et al. [97] evaluated polyelectrolyte complexes of TMC and some polyanions (hyaluronan, alginate and dextran sulfate) in their gene delivery efficiency to MCF7 cells through an experimental design. In vitro studies showed non-toxicity and high cellular uptake of the nanoparticles. In vivo studies indicated significant tumor uptake with low accumulation of nanoparticles in vital organs such as heart, liver and lungs. Such findings suggest that TMC nanocomplexes can be an efficient and safe gene delivery system, especially for cancer therapy.

Polymeric systems, especially composed by chitosan, TMC or other quaternary compounds, have displayed great potential to be used as adjuvants (agents that improve the immune response) for vaccines. As reported by Slütter et al. [98], such materials can improve interaction of antigens with dendritic cells and induce their maturation, thus increasing the probability of antigen uptake. Most of the studies that applied TMC in vaccines involved nasal administration, with improved delivery of inactivated influenza virus [99,100], tetanus toxoid [101], diphtheria toxoid [102], peptide antigens [103], recombinant proteins [104] and a series of immunopotentiators [105]. Trimethyl chitosan has also increased immune responses in intradermal [105,106] and intraperitoneal [107] vaccination.

Another application in which chitosan is widely studied is wound dressing. However, very few studies have attempted to use TMC as a component of wound healing materials. Zhou et al. [108] synthesized TMC fibers that presented higher absorption capacity and higher antibacterial properties against *E. coli* and *S. aureus* than chitosan fibers. Such material exhibited no cytotoxicity to mouse embryo fibroblast cells and in vivo experiments resulted in excellent wound healing activity in full-thickness excision wound model in rats. Patrulea et al. [109] synthesized O-carboxymethyl-N,N,N-trimethyl chitosan (CMTMC), which was then functionalized with Arg-Gly-Asp-Cys peptide. The system was used in three different formulations: hydrogels, foam-like patches and NP suspensions to be sprayed onto wounds. The produced materials were able to preserve peptide bioactivity, allowing complete wound closure in an in vitro assay. Such approach still needs further in vivo evaluation.

The use of trimethyl chitosan in tissue engineering was also barely evaluated. Due to its biocompatibility and non-toxicity, chitosan and its derivatives have shown high potential to compose biomimetic systems. Romero et al. [110] produced polyelectrolyte multilayers of TMC-heparin on cortical bone allografts, either bare or coated with chitosan. Such scaffolds demonstrated potential to mimic the dense fibrous membrane covering the surfaces of bones, also known as periosteum. The authors reported that all the samples were cytocompatible with adipose-derived stem cells. Additionally, the surfaces were able to incorporate osteoprogenitor cells and supported an osteoprogenitor cell phenotype without driving cell differentiation.

While chitosan-based sensors have been featured in several studies, TMC-based sensors have barely been studied. Zhao et al. [78] synthesized fluorophore-labeled TMC nanoparticles with an average diameter of 323 nm and a positive zeta potential as large as 37.1 mV, which characterizes such NPs to be used as carriers for crossing tissue barriers. A DNA aptamer which can specifically bind to *E. coli* was then covalently bound to TMC’s surface. The authors consider that such type of system offers an efficient tool to understand the pathway of bacteria crossing the host cell barrier to fight against invasive infections.

In respect to environmental applications, some studies have evaluated the used of TMC in wastewater treatment. Mohammad et al. [43] loaded cerium oxide particles with trimethyl chitosan and assessed the biological activity and biosorption performance of the composite. The authors reported significant enhancement of antibacterial and antioxidative efficiency of CeO${}_{2}$ because of its increased interaction with bacterial cell wall of *E. coli* and *S. aureus* promoted by the TMC coating. They also observed improved biosorption of phenolic compounds such as phenol, 2-chlorophenol and 4-chlorophenol when compared to that of cerium oxide by itself. Tabriz et al. [111] synthesized TMC and incorporated it in polyethersulfone (PES) polymer to fabricate membranes for enhanced antifungal activity and drinking water treatment. They found out that an increasing content of TMC in the membranes resulted in improved properties such as porosity, hydrophilicity, permeance flux and surface roughness. In addition, the membranes with the higher content of TMC (15%) were the ones with the most antifungal activity against *Fusarium solani*. These findings support the potential application of trimethyl chitosan in wastewater treatment systems as filtration beds and membranes.

One of the biggest concerns of mining companies is waste disposal, which is normally done in the form of tailing dams. Such approach presents operational risks and the effluent that composes the dam must be treated in order to fit legal parameters. In the aluminum production process, for example, bauxite beneficiation is one of the steps that generate wastes, which is normally treated through coagulation with the use of aluminum sulfate in order to reduce the turbidity levels before its return to the environment. In this context, Bigogno et al. [112] synthesized TMC and evaluated its use as a coagulant in bauxite treatment for tailing dam effluent in comparison to protonated chitosan and conventional aluminum sulfate. The authors reported that TMC presented higher reduction of effluent turbidity in the same experimental conditions, showing potential to be used in such type of application.

Abu Elella et al. [113] developed an adsorbent to simultaneously act as a filter for capturing crystal violet dye and inhibiting the growth of *E. coli*. This was achieved with the use of interpenetrated polymer network films composed of TMC and xanthan gum (XG) that were prepared by casting method. By optimizing the conditions of the preparation process, the authors were able to remove 94.4% of the dye. In addition, the films inhibited bacterial growth in all the experimental conditions, showing potential to be used as antibacterial adsorbents in wastewater treatment.

Regarding food industry, chitosan coatings have been extensively studied due to its bioactivity and excellent film-forming properties. As a result of the toxicity presented by the methylation agents used to produce TMC, the compound has not been very studied in such applications. However, De Britto and Assis [39] evaluated the use of chitosan and some of its quaternary derivatives, such as TMC, as active coatings for sliced apples. As a result, the coatings displayed lower browning effect on cut apple surface when compared to the uncoated samples. In addition, TMC showed significant antifungal activity against *Penicillium expansum* and moderated inactivation of *Botrytis cinerea*.

## 3. HTCC

### 3.1. Structure and Properties of HTCC

**Figure 3:**
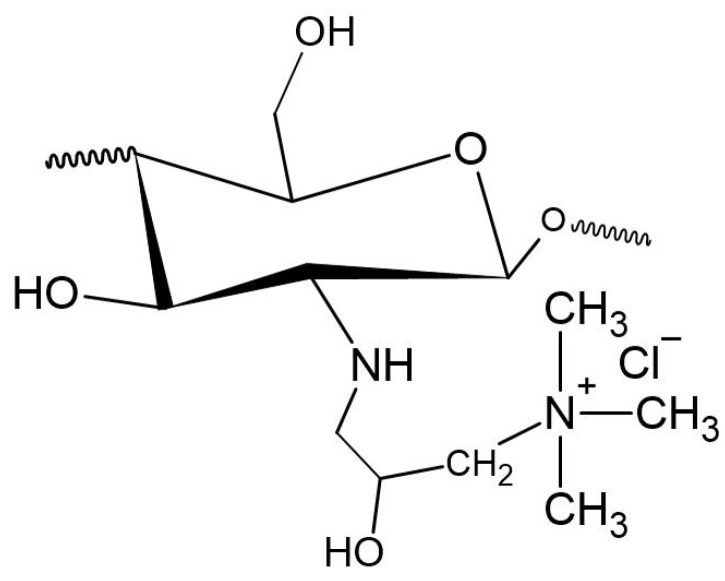
Molecular structure of N-(2-hydroxyl)propyl-3-trimethyl ammonium chitosan chloride (HTCC).

The second most common route for quaternizing chitosan is through the introduction of a quaternary ammonium moiety outside the chitosan backbone by an alkylation reaction, leading to the formation of N-[(2-hydroxy-3-trimethyl ammonium) propyl] chitosan, or HTCC, which has chloride as counter-anion and whose structural formula is shown in Figure 3 [20].

The first reports related to the production of HTCC date from the 1980s, when Lang et al. [114] reported on the chitosan modification, without however presenting results of characterization of derivatives, which was later performed by Loubaki et al. [115], who carried out elemental analysis and infrared spectroscopy, in addition to ${}^{13}$C- and ${}^{1}$H-NMR spectroscopy. Initially, the focus of HTCC synthesis was on the cosmetic market, having generated several patents at that time [33]. However, an important branch of application of HTCC related to antimicrobial action has gained attention in recent decades, having been firstly reported by Kim et al. [29], who applied it as an antimicrobial finish of cotton fabrics.

HTCC is also a cationically modified chitosan, considered thus by the presence of ammonium groups that are ionized regardless of pH value [116]. Its most common synthesis route is through the contact of chitosan with glycidyl trimethylammonium chloride (GTMAC), which can generate HTCC with different DQs depending on stoichiometry [115]. The DQ refers to the number of positively charged quaternary ammonium groups present in the chitosan chain and according to Seong et al. [117], the reaction time and temperature, and the ratio of GTMAC to chitosan can affect its value. The reaction mechanism for producing HTCC is explained by the attack of chitosan amino groups on the C atom with the least steric hindrance of the GTMAC molecule, which is thermodynamically and kinetically more favorable than the attack by the chitosan hydroxyl groups [118].

Biologically, HTCC presents characteristics very similar to chitosan, such as non-toxicity, biodegradability, biocompatibility, mucoadhesiveness and antimicrobial activity. Nevertheless, it also has some unique valuable properties that are not attained in the non-modified polymer [116,119].

According to Chen et al. [120], the addition of quaternary ammonium to chitosan was capable of improving its water-solubility, antibacterial activity and biocompatibility. The increase in water solubility may be justified by the weakening of the hydrogen bonds in the ordered arrangement of chitosan due to the introduction of quaternary ammonium compound, which causes an increase in the charge strength. Shagdarova et al. [121] showed that HTCC presented more pronounced antibacterial, antioxidant and antifungal properties than the non-modified chitosan. The improvement in antibacterial activity may be related to the positively charged groups present in HTCC structure, which interact better with the negatively charged structures on the cell surface.

Hecq et al. [38] reported that the mucoadhesive property, characterized by the positively charged surface which favors electrostatic interactions with the enteric surface, with negatively charged mucins, has been improved in HTCC in relation to chitosan. Chitosan had a positively charged surface only in acidic medium, while HTCC was less sensitive to pH variation, with a positively charged surface even at neutral pH. Mucoadhesion is one of the key factors for polymers applicable as absorption enhancers on mucosal surfaces. To confirm the possibility of its use as a drug carrier or for food processing, for example, it is important to assess its toxicity in vivo. Wang et al. [122] evaluated the effect of HTCC in vivo, introducing the compound in the diet of mice for 30 days. The trials did not detect toxicity immediately or after the HTCC treatment period, nor mortality under the same conditions. No changes in appetite were observed, either in feces or behavioral nor in body weights. The authors detected a reduction in the levels of Fe, Zn and Ca in the mice livers.

Xiao et al. [123] compared the crystallinity of HTCC and chitosan, concluding that HTCC has a more amorphous structure, consistent with its greater solubility. This means that quaternization induces the breakdown of inter- and intra-molecular hydrogen bonds, causing the breakdown of the crystalline structure observed in the non-modified polymer [124]. This change in the crystalline structure and the formation of a looser structure, also helps to favor the diffusion of free water molecules in the molecular chains of HTCC, resulting in a better ability to absorb liquids [125].

HTCC properties are usually influenced by the DQ used. Wang et al. [126] evaluated the rheology of the material and found that both the apparent viscosity and the modulus of elasticity, or storage, (G’) increase with the increase of the DQ. In addition, the authors concluded that the material had a predominant elastic property, with the formation of a gel-like structure. Shagdarova et al. [121] determined that the water solubility of HTCC was improved with an increase in DQ, as well as its antibacterial activity against microorganisms of *Staphylococcus epidermidis* and *Escherichia coli*. Wang et al. [127], aiming at the application of HTCC as an adjuvant in vaccines, determined that lower DQs resulted in greater hydrophobicity and unoccupied amino groups and, consequently, greater ability to adsorb antigens through hydrogen bonds.

Regarding the antibacterial activity of HTCC, it can occasionally be uncertain. Seong et al. [117] and Kim et al. [128] confirmed the increased antibacterial efficiency of HTCC in relation to chitosan against a Gram-negative bacterium, *Escherichia coli*, and a Gram-positive bacterium, *Staphylococcus aureus*. Chi et al. [129] found that HTCC showed a biocidal characteristic against Gram-positive bacteria, but not against Gram-negative bacteria. In addition, the authors noticed an effect of pH, with microbiocidal activity being slightly stronger in alkaline conditions than in weak acidic conditions, a fact also observed by Qin et al. [130].

Hoque et al. [131] evaluated the antibacterial and antifungal efficacy of HTCC against multi-resistant bacteria and pathogenic fungi. Discovering an effective agent against fungi, that does not present harmful effects to man, is a challenge due to the eukaryotic characteristic of both species, increasing the need for a selective action agent against fungi. In addition, the proliferation of fungi can cause an increase in disease severity due to the association with bacterial infections. The authors tested HTCC against different strains of bacteria, sensitive or resistant to drugs, Gram-positive or Gram-negative. In general, HTCC had both bacteriostatic and bactericidal activity against all tested bacteria, being more active against Gram-positive bacteria than against Gram-negative bacteria, which proves its broad spectrum nature. The tests also showed that the bacteria had difficulty in developing resistance to the action of the polymer, differently when they are in contact with traditional antibiotics. In relation to antifungal activity, HTCC inhibited the growth of most human pathogenic fungi, with fungistatic and fungicidal action. Both the antibacterial and the antifungal action occurred through the interaction of the cationic polymer with the negatively charged cell membranes, causing their disruption and consequently cell death. In addition, HTCC demonstrated non-toxicity to mammalian cells in vitro and to rat skins in vivo.

Due to the widely studied antimicrobial activity of HTCC, Milewska et al. [132] also evaluated its potential antiviral activity. The authors verified the efficiency of HTCC in preventing the replication of human coronaviruses (HCoVs), but without inhibitory activity against other viruses, showing its highly specific behavior. In addition, HTCC did not show cytotoxicity, making it a promising material for applications as a treatment agent. Milewska et al. [133,134], in turn, determined the mechanism of antiviral action of HTCC, which occurred through the interaction between HTCC and the coronavirus’ S protein, blocking its interaction with the cell receptor and, thus, hampering viral infection. Furthermore, the authors established that HTCC with different DQs were effective against different HCoVs, suggesting that this material can be optimized to become active against other species.

Depending on the purpose of the HTCC application, it can be combined with other compounds to improve its characteristics or acquire new ones. Lee and Kim [135] successfully developed grafted copolymers of HTCC and carboxyl group-terminated poly (N-isopropylacrylamide) (PNIA-COOH) to obtain a thermo-responsive material and increase its commercial application, as HTCC does not have this property. The lower critical solution temperature of the copolymer, above which phase separation occurs, was slightly higher than that of pure PNIA. Wu et al. [136] performed the quaternization of chitosan and poly (ε-caprolactone) copolymers with GTMAC. The authors found that this material can be well used in antibacterial applications that require high mechanical strength in the wet state, a characteristic that is not achieved in chitosan membranes that have greater mechanical strength in the dry state.

Mivehi et al. [137] prepared films and fibers from the blend of HTCC and polyacrylonitrile (PAN), which were subjected to dyeing steps. The interaction between HTCC and PAN was strong enough to prevent the former from being solubilized in boiling water and, in addition, the dyeing behavior was improved in the blend. The increase in the amount of HTCC in the blend contributed to better antimicrobial activity and better tensile strength.

### 3.2. Preparation Methods

**Figure 4:**
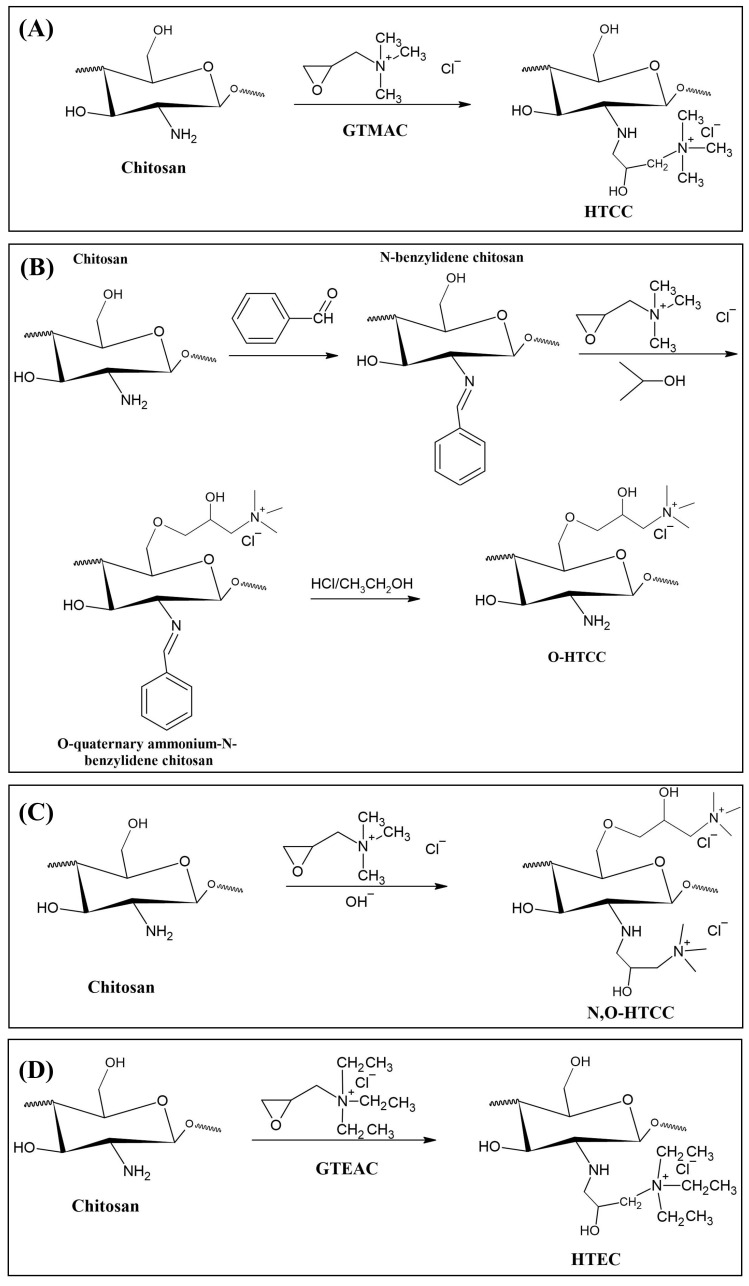
Reaction route for the synthesis of (**A**) HTCC; (**B**) O-HTCC; (**C**) N,O-HTCC and (**D**) N-(2-hydroxyl) propyl-3-triethyl ammonium chitosan chloride (HTEC), from chitosan.

The most common and most widely studied method for obtaining HTCC is by the reaction between chitosan and GTMAC, according to the synthesis route shown in Figure 4a. In general, this reaction is more favored under acidic conditions, which makes the epoxy group more reactive and easier to open. Furthermore, under acidic conditions the epoxy group reacts more favorably to NH_2_ groups of chitosan, while under basic conditions, the reaction by the OH groups of chitosan is more favored [22].

Most of the studies that used the aforementioned methodology either performed it in an aqueous medium or used acetic acid as a reaction catalyst. Therefore, chitosan is dispersed in deionized water (and acetic acid, when applicable). GTMAC aqueous solution is added in different amounts to obtain different DQs. In general, the acid-catalyzed reaction is conducted over a 10–18 h period, at temperatures ranging from 50–80 °C [22,35,117,120,138]. In cases where the catalyst is not used, a longer time (up to 24 h) or a higher temperature (85 °C) may be necessary to achieve expected results [21,29,130,139]. As reported by Seong et al. [117], who studied the influence of the time and temperature parameters on the DQ, the DQ increases with increasing temperature and reaction time, up to an optimum, in their case 80 °C and 18 h, with little variation in DQ values above these conditions.

It is also possible to find more specific cases of the reaction of chitosan and GTMAC. Lu et al. [140], Wu et al. [141] and Zhou et al. [125] carried out the reaction by dissolving chitosan in isopropanol, with a reaction time in the range of 7–10 h, at a temperature of 80 °C. Nam et al. [142] used Zn(BF_4_)_2_ as a catalyst for the reaction, with elevated time (20 h) and temperature (100 °C) conditions, and Ruihua et al. [143] dispersed chitosan in deionized water and perchloric acid, the reaction occurring at 80 °C for 8 h. As noted, HTCC synthesis is normally carried out in a heterogeneous reaction medium and involves several types of volatile organic reagents or other aggressive chemical reagents. To overcome these issues, Yang et al. [118] proposed the employment of an ionic liquid of 1-allyl-3-methylimidazole chloride (AmimCl) as a green and homogeneous reaction medium. Thus, the chitosan was dispersed in AmimCl until complete dissolution. GTMAC was added and maintained in reaction for 8 h at 80 °C.

The second most used route for the production of HTCC is through the reaction between chitosan and 3-chloro-2-hydroxy-propyl trimethyl ammonium chloride (CTA), a lower cost reagent than the usual GTMAC [9]. CTA is used as an etherification reagent due to its characteristic of generating an epoxide in alkaline conditions, which is similar to GTMAC [144]. In general, chitosan is dispersed in isopropanol or 2-propanol, over which NaOH solution is added and kept under stirring for a time that can vary from 2–5 h. After this step, aqueous CTA solution is added and the reaction can be conducted under conditions ranging from 6–10 h and 40–60 °C [145,146,147]. Ali and Singh [148], in turn, dispersed the chitosan in water, requiring reaction conditions of 33 °C and 18 h, a significantly longer time than that performed by authors who used other solvents. Tan et al. [149] dispersed chitosan in an alkaline aqueous solution of LiOH/KOH/urea/H_2_O, which is considered an environment-friendly solution. Later, they added CTA solution dropwise to the chitosan solution, the reaction taking 12 h to complete.

In addition to HTCC synthesis, some studies report the synthesis of other compounds similar to HTCC, such as O-HTCC (O-(2-hydroxyl) propyl-3-trimethyl ammonium chitosan chloride), characterized by the insertion of quaternary terminals in the hydroxyl group of chitosan. This reaction also occurs through the contact of chitosan with GTMAC, though under conditions where the NH${}_{2}$ functional groups are protected [150]. The procedure for obtaining the O-HTCC usually proceeds through three steps: the first to protect the NH_2_ functional group, the second to insert the quaternary terminal into the hydroxyl group, and the third to release the NH${}_{2}$ functional group. In general, chitosan is dispersed in acetic acid, to which benzaldehyde or any benzoyl hydride is added to protect the NH_2_ group, producing N-benzylidene chitosan. After neutralization with NaOH, solution of GTMAC dispersed in isopropyl alcohol is added and maintained in reaction for 16 h at 70 °C. The product generated in this step is added to an ethanolic HCl solution, obtaining crude O-HTCC, which must be purified in suitable solvents [150,151,152]. Figure 4b presents the synthesis route aforementioned [153].

Additionally through the contact of chitosan with GTMAC, it is possible to obtain a third product, N,O-HTCC (N,O-[(2-hydroxyl-3-trimethyl-ammonium) propyl] chitosan chloride), obtained from an additional quaternization of HTCC through hydroxyl groups, under alkaline conditions, according to Figure 4c. The production of N,O-HTCC is possible due to the elevated reactivity of the hydroxyl groups at high pH values, leading to substitutions at both N and O units. The addition of this step is able to improve the DQ and generate products with a high charge density. However, care must be taken that the high density of ammonium groups does not cause such repulsion between them, to the point of reducing antibacterial activity [154,155,156].

Another compound that was also obtained by Wan et al. [157], similar to HTCC, is N-(2-hydroxyl) propyl-3-triethyl ammonium chitosan chloride (HTEC), which was obtained by the reaction between chitosan and glycidyl triethylammonium chloride (GTEAC), according to the synthesis exposed in Figure 4d. HTEC was obtained by dispersing chitosan in distilled water at 85 °C. After the addition of GTEAC, the reaction proceeded for 10 h, a condition similar to most of the synthesis routes presented for HTCC. Comparing the DQ values of HTCC and HTEC obtained by the authors, it was observed that HTCC has a higher DQ, possibly because GTEAC has a higher steric hindrance characteristic, reacting less than GTMAC with chitosan.

### 3.3. Applications of HTCC and Similar Compounds

As HTCC has been extensively studied in recent decades, it finds applications in several fields, especially for its advantageous properties such as water solubility, enhanced permeability due to hydration, mucoadhesivity, antibacterial activity and strong steric hindrance of positively charged quaternary groups, in addition to properties from unmodified chitosan, such as biocompatibility and biodegradability [123]. As mentioned, the primary development of HTCC aimed at its application as a cosmetic agent. The interaction of the quaternary ammonium group with the anionic hair groups made it possible to use these compounds as a conditioning agent for hair treatments [33].

Given HTCC’s antibacterial activity, Kim et al. [29] evaluated its application as an antimicrobial cotton finisher. The authors found that HTCC-treated cotton presented almost 100% bacterial reduction, even at low concentrations. However, due to the aqueous solubility of HTCC, its durability as a cotton finisher could be limited by the launderings number. Thus, the authors reported that the conjugation of HTCC with a commercial nonionic binder was able to provide good laundering durability in terms of antimicrobial activity. Kim et al. [128] also evaluated the microbial activity of HTCC-treated cotton fabrics. However, an appreciable antimicrobial activity was not observed, even after a single laundering. Thus, polycarboxylic acids were added to improve the immobilization of HTCC, obtaining bacterial reductions greater than 91% after 20 launderings, without affecting the mechanical strength or whiteness of the fabric.

Zhu et al. [30], taking advantage of the antimicrobial property of HTCC, evaluated its use in the control of cyanobacteria blooms, which occur due to the high load of nutrients and pollutants in the water and can generate cyanotoxins capable of threatening aquatic and human life. The authors found that HTCC with a DQ of 98% was capable of inhibiting the growth of *M. aeruginosa*, the most common harmful algae species, and reduce the release of cyanotoxins. Possibly, HTCC acts by destroying the cellular structure of these cyanobacteria that are related to photosynthesis. Jin et al. [158], following the same research field, proved the possibility of using HTCC as a coagulant in drinking water treatment to remove *M. aeruginosa*. Vanitha et al. [159], in turn, evaluated the larvicidal action of HTCC against larvae in aquatic bodies of two species of mosquitoes, *Culex* and *Aedes*, vectors of a large number of infectious diseases. When compared to the action of silver nanoparticles, HTCC demonstrated equivalent larvicidal activity, with the advantages of biodegradability, biocompatibility and non-toxicity. The positive charge of HTCC electrostatically interacts with the negative surface residues of the microbial membranes, killing the mosquito larvae.

The vast majority of research involving HTCC covered its biomedical and pharmaceutical application due its biocompatibility, mucoadhesiveness, water solubility, etc. Mi et al. [160] and Mi et al. [34] produced porous chitosan microspheres by coagulation in an aqueous solution of TPP (tripolyphosphate) and subsequent chemical modification by adding quaternary ammonium groups through CTA reagent. The authors’ objectives were, respectively, to obtain a means of controlled release of Newcastle disease vaccine antigen and a new delivery system for the indomethacin drug. Both the antigen and the indomethacin were subjected to adsorption in quaternized and unmodified chitosan, the former having the highest adsorption capacities due to the electrostatic attraction between the quaternary ammonium group and the negative charges of the two compounds. Xu et al. [161] also evaluated HTCC nanoparticles ionically gelled with TPP, aiming to evaluate its application as a protein carrier, in this case, bovine serum albumin (BSA) as a model protein drug. The authors found that HTCC nanoparticles were a potential vehicle for protein administration, with a high capacity for incorporation and the possibility of prolonging release. This result was expanded by Zhao et al. [162], who successfully produced HTCC and TPP nanoparticles for incorporation of Parathyroid Hormone-Related Protein (PTHrP), a polypeptide capable of promoting bone formation and proliferation of osteoblasts, but which is expensive and easily denatures, requiring a system of controlled release.

Another important application of HTCC was reported by Kaminski et al. [35] and refers to the action as a reversal agent for heparin, an anticoagulant extensively used but which, in emergency situations, may need to be stopped immediately. HTCC (DQ = 90.5%) appears as an option to the usual protamine sulfate, which can cause several adverse effects and low efficiency of action on heparin. The results showed that HTCC binds satisfactorily to low- and high-molecular-weight heparins at a typical blood pH. In addition, HTCC forms smaller aggregates with heparin and with less polydispersity, which can be advantageous in medical procedures, such as intravenous applications. Lorkowska-Zawicka et al. [163] evaluated the aforementioned results with applications in rats in vivo and in rats’ blood in vitro, and observed that the reversal action of heparin is maintained under the conditions evaluated, safely and without toxic effects when administered in doses adjusted for complexation with heparin.

Xiao et al. [123] reported the application of HTCC in gene delivery, a promising therapy in the treatment of inherited or acquired diseases. The fact that these genes have a low plasma half-life demands an adequate system for their transfection. As a result, the authors found that HTCC with a DQ in the range of 12.4–43.7% is an excellent candidate as a non-viral vector, showing high efficiency of in vitro gene transfection, with negligible cytotoxicity. Still considering its biomedical application, HTCC was also evaluated as vaccines adjuvant or delivery platform. Wu et al. [141] developed a nasal vaccination system, aiming to take advantage of HTCC bioadhesiveness for the Zaire Ebola virus antigen delivery. By developing a thermosensitive hydrogel with HTCC, it would be possible to extend the residence time of the antigen in the nasal cavities and, consequently, its penetration through the nasal mucosa. The advantage of HTCC is that it binds strongly to the negatively charged mucosal surface and can also loosen the strong conjugation between epithelial cells, allowing greater antigen action. As a result, the use of the HTCC-containing hydrogel to deliver the antigen stimulated immune responses from the respiratory mucosa, with broad humoral immunity and low toxicity to nasal tissues and cells, and better results for moderate quaternized HTCC (DQ of 60% and 79.5%). The action as a vaccine adjuvant was evaluated by Tao et al. [147], aiming to immuno-intensify the action of the hepatitis E virus antigen, and by Wang et al. [127], for the H5N1 avian influenza virus antigen. In the case of the latter, it is interesting to add adjuvants due to the low immunogenicity of vaccines, especially in very young or elderly populations. According to the authors, HTCC was able to induce a strong immune response in a safe and effective manner, and may be an alternative to the commonly used aluminum that has a low capacity to stimulate immune responses.

Zhou et al. [125] described the use of HTCC as a wound dressing, especially for its high antibacterial activity and high fluid absorption capacity, favoring healing and eliminating the possibility of infections and trauma in the patient by changing dressings. Tests with *S. aureus* bacteria, the most common pathogen responsible for secondary infections in wounds, indicated high antibacterial activity by HTCC on this bacterial strain. To confirm the use in wound dressings, the authors indirectly verified that HTCC does not exert cytotoxicity in vitro on mouse fibroblasts.

In addition to biomedical applications and as antibacterial agents, other specific research fields involve HTCC, due to its unique properties. The adsorption capacity of HTCC has been extensively studied for several applications, such as for the treatment of effluents from the textile industry, rich in reactive dyes. Rosa et al. [164] used HTCC for adsorption of the reactive dye orange 16 and obtained a pH-independent process, with a maximum adsorption capacity of 1060 mg per gram of adsorbent, equivalent to the occupation of 75% of the adsorptive sites. The pH independence is due to the interaction of the HTCC ammonium groups with the sulfonated groups of the dye. Spinelli et al. [139] reported adsorption of Cr(VI) in HTCC, which proved to be pH dependent. The maximum adsorption capacity was 68.12 mg per gram of adsorbent, corresponding to 62.7% of occupation of the active sites. In addition, it was found that HTCC can be regenerated in adsorption/desorption cycles to be reused as an adsorbent, making the process more economical.

Zhang et al. [165], also based on the adsorptive capacity of HTCC, evaluated the adsorption of proteins as an enrichment/bioseparation step in the production of these compounds. The authors were able to successfully obtain soybean peroxidase from crude soybean peroxidase solution using HTCC. Still using the adsorptive capacity as a step of separation, concentration and purification, Ciejka et al. [166] proposed HTCC as an adsorbent for coronaviruses (human HCoV-NL63 and HCoV-OC43 and mouse MHV), aiming at obtaining them for the preparation of products in which the virus is the main component (e.g., research purpose, vaccines manufacturing or gene therapy). HCoV-NL63 and MHV were successfully adsorbed, while HCoV-OC43 did not show significant adsorption. In the case of NL63, advantageously, selective adsorption was observed, in addition to the possibility of desorption of the virus that retains its virulence when desorbed.

Li et al. [167] evaluated the application of HTCC in the papermaking process to replace a commercial cationic starch. HTCC was added as a retention-aid, that is, a flocculating agent for the natural occurring CaCO${}_{3}$, a filler used in alkaline papermaking processes and that gives the paper shine, whiteness, opacity and good printability. The authors found that HTCC adsorbed almost completely on the surface of CaCO${}_{3}$, due to electrostatic interactions between the surface of the positively charged quaternized chitosan and the negative charges on the surface of the CaCO${}_{3}$ particles. In addition, HTCC-induced flocculation was much more pronounced than cationic starch flocculation. In addition to its use as a flocculant, Li et al. [168] also evaluated HTCC as an additive to improve the physical and mechanical properties of paper and its manufacturing process. The adsorption of HTCC on cellulosic substrates was stronger than that of cationic starch and required less optimal concentration to achieve the expected results due to differences in molecular configuration and charge densities.

An additional use evaluated for HTCC was as a membrane in alkaline fuel cells, a promising alternative to future energy needs. Compared to chitosan, which, when hydrated, presents characteristics of ionic conductivity, HTCC can function as a gel rich in positive charges, presenting potential for the production of anionic exchange membranes. HTCC membranes cross-linked with glutaraldehyde, and added with NaBH${}_{4}$ to improve their stability and mechanical resistance, were produced. These membranes demonstrated high ionic conductivity, compared to that of Nafion N117, a commercial membrane widely used, but which presents restrictions in the temperature range and high cost. Such restrictions can be eliminated with the use of quaternized chitosan [169]. Still in the field of energy research, HTCC was used as a shale inhibitor in drilling fluid, aiming at increasing the effectiveness and efficiency of shale oil/gas drilling in a scenario of increasing energy demand and decreasing conventional oil and gas reserves. The use of environmental friendly HTCC comes as an alternative to the increasingly strict regulations on the use of oil-based drilling fluids and has the advantage of positive groups that are able to bind to the negative groups of clay particles. The results demonstrated that HTCC inhibits shale better than the usual polyether amino, with greater resistance to temperature [170].

Within HTCC’s extensive list of applications, it is possible to combine it with other compounds, such as the aforementioned TPP, further expanding its application possibilities. Grant et al. [171] developed an injectable biocompatible formulation, composed of the blend of HTCC, egg phosphatidylcholine (ePC) and fatty acid chlorides with different lengths of the acyl chain, for localized release of paclitaxel, an anti-cancer agent for the treatment of solid tumors. The authors were able to obtain a perfectly injectable system and sustained release over a three-week period. Tan et al. [172] combined HTCC (DQ = 26%) and poly-methylmethacrylate (PMMA), a base for acrylic bone cement, to inhibit the production of bacterial biofilms on the bone cement surface in order to prevent and treat common bacterial infections in surgical orthopedic procedures. HTCC-PMMA demonstrated strong inhibition against strains of *S. epidermidis* and drug-resistant *S. aureus*, having a more pronounced effect than PMMA, chitosan-loaded PMMA and the commonly used gentamicin-loaded PMMA. Huang et al. [173] reported HTCC and fucoidan nanoparticles (NPs) to carry epigallocatechin gallate (EGCG), a tea polyphenol with antioxidant and hypoglycemic action, extremely sensitive to heat and oxidation reactions, and which has low absorption in the body. By loading it into the NPs, the authors were able to protect the compound from degradation, control its release and even increase its absorption. In addition, the EGCG-loaded NPs presented improved antioxidant and antibacterial action and contributed with stronger inhibition of digestive enzymes, being a method of preventing diabetes mellitus.

## 4. Quaternized Chitosan with Pyridinium Salts

**Figure 5:**
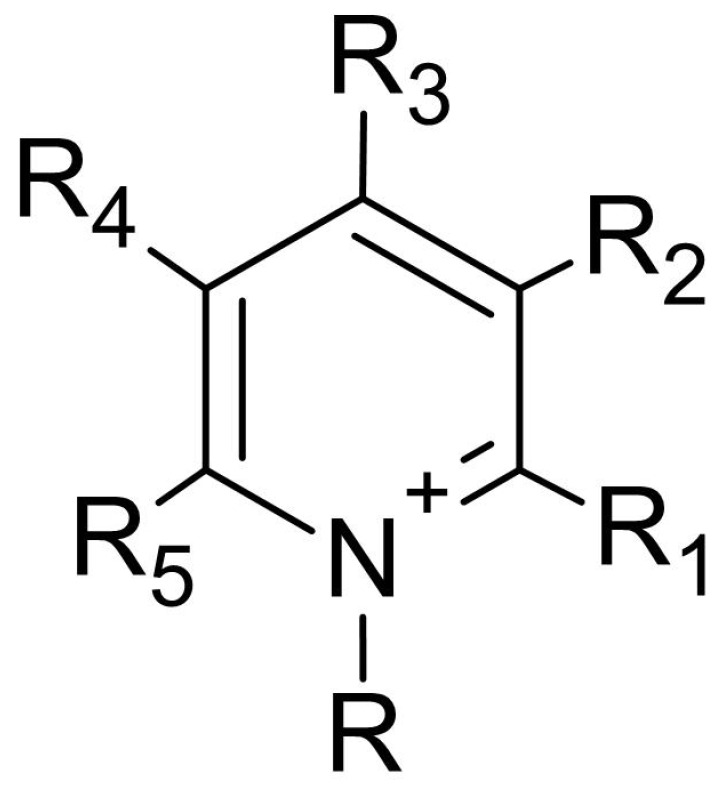
Pyridinium ring.

Pyridinium salts constitute a class of unsaturated heterocyclic molecules (Figure 5) and the main route of obtaining them is through SN_2_ type reactions between pyridine and alkali halides [174,175,176].

Pyridine derivatives were introduced into the polymer backbone to improve polymer properties including solubility, physicochemical and biological properties [63]. Several studies have proven that pyridinium salts have a wide range of applications, including antiviral, antibacterial and antifungal activity [177,178]. According to Sowmiah et al. [179], pyridinium salts are known to inhibit the growth of various microorganisms, such as bacteria, viruses and fungi. This inhibition occurs through the cationic groups present in the pyridinium that are attracted by negatively charged microorganisms, resulting in disorganization and denaturation of proteins in the microorganism membrane and, thus, causing cell death [15].

**Figure 6:**
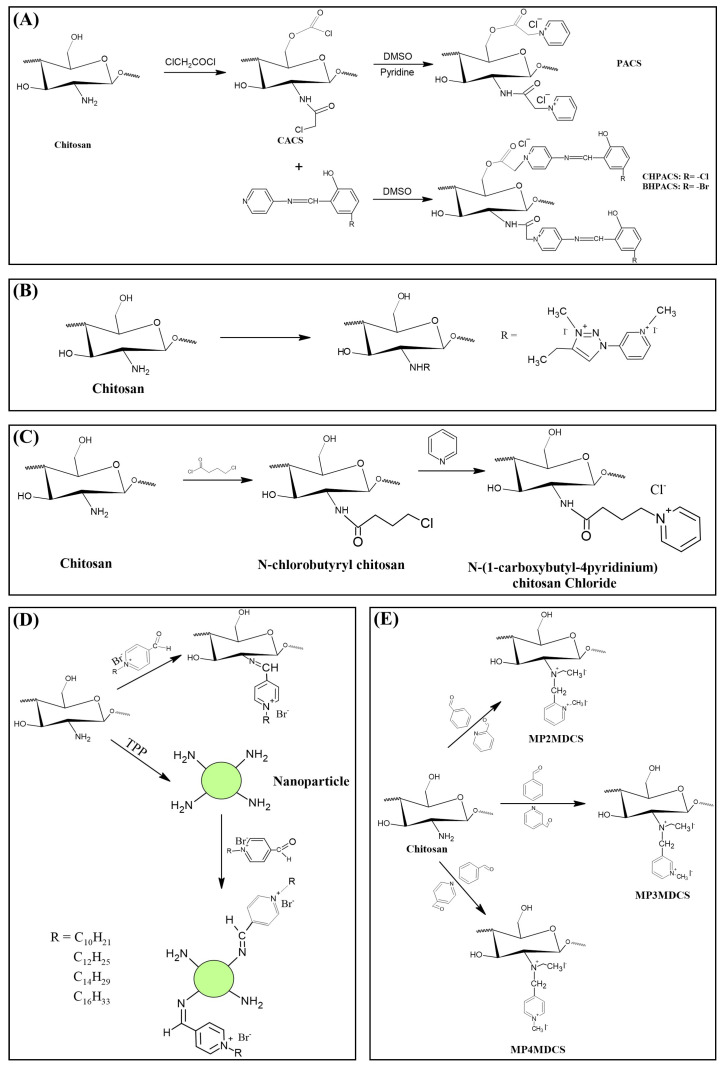
Synthetic route to obtain different quaternized chitosan derivatives with pyridinium salts: (**A**) PACS, CHPACS and BHPACS, (**B**) chitosan derivative containing N-methyl-1,2,3-triazoliumand N-methyl-pyridinium, (**C**) N-(1-carboxybutyl-4-pyridinium)chloride chitosan, (**D**) CS and NP chitosans and (**E**) MPMDCSs.

The first study on the incorporation of pyridinium salts in chitosan was reported in 2010 by Li et al. [180]. The authors presented three new quaternary chitosan derivatives, called PACS, CHPACS and BHPACS, synthesized from the reaction between chloroacetyl chitosan (CACS) and pyridine, as shown in Figure 6a, and tested the antifungal activity of both against four pathogenic fungi, namely, *Cladosporium cucumerinum*, *Monilinia fructicola*, *Colletotrichum lagenarium* and *Fusarium oxysporum*. The CHPACS and BHPACS derivatives were synthesized from a solution of 5-chlorosalicylaldehyde or 5-bromosalicylaldehyde and the product of this reaction was added together with CACS. The authors observed that the derivatives CHPACS and BHPACS had an inhibitory effect against pathogenic fungi superior to that presented by the other derivatives and the chitosan itself, presenting an effect of 100% in concentrations of 500 μg/mL and 1000 μg/mL which happened by the presence of groups 5-chloro-2-hydroxybenzylideneamino and 5-bromo-2-hydroxybenzylideneamino in the structure of the derivatives. This fact was also reported by Guo et al. [181] in their work, where they noticed an increase in the inhibitory effect of the chitosan derivative when the group 5-chloro-2-hydroxybenzylideneamino was inserted in the chitosan structure.

Tan et al. [23] developed a cationic derivative of chitosan containing N-methyl-1,2,3-triazolium and N-methyl-pyridinium via efficient cuprous-catalyzed azide-alkali cycloaddition reaction (CuACC). The derivative was synthesized in three stages: initially, the cationic derivative of propargyl chitosan (a) was synthesized from the reaction of chitosan with propargyl bromide; then, derivative (a) was dissolved in a DMSO solution and 3-azidopyridine, triethylamine and cuprous iodide were added, producing the cationic derivative containing 1,2,3-triazole and pyridine (b); finally, derivative (c), containing 1,2,3-triazolium and pyridinium was synthesized from N-methylation, reacting derivative (b) with iodomethane. Figure 6b shows a summary of the synthesis of compound (c). The authors tested the antifungal potential of the derivative with three phytopathogenic fungi, *C. lagenarium*, *W. fusarium* and *F. oxysporum*. The derivative containing 1,2,3-trizolium and pyridinium in its structure showed a superior antifungal action when compared to the other derivatives, presenting a maximum inhibition rate of 98.44% for *C. lagenarium*, followed by 79.16% and 67.56% for *W. fusarium* and *F. oxysporum*, respectively, at 1.0 mg/mL, showing a potential antifungal agent. In addition, it has better solubility in water, especially at alkaline pH.

Jia et al. [24] introduced, by nucleophilic substitution, portions of pyridine to obtain N-(1-carboxbutyl-4-pyridinium) chloride chitosan. The synthesis process (Figure 6c) of this new derivative took place through an initial treatment of chitosan with 4-chlorobutyl chloride for the production of N-chlorobutyl chitosan, which then reacted with pyridine, finally forming a chloride of quaternary ammonium, N-(1-carboxybutyl-4-pyridinium) chitosan. The antifungal activity of this derivative was tested with *B. cinerea* and *F. fulva*, where the antifungal index of pyridine chitosan was 75%, while that of chitosan was only 58.9%, in addition, serious morphological changes of *B. cinerea* when treated with pyridine chitosan, where it caused the damage and deformation of the structure of fungal hyphae and, subsequently, the inhibition of the growth of the strain. A similar fact was observed by [182], where the positively charged portion of the cationic molecule directly interfered with the fungal cell surface, changing the permeability of the plasma membrane and thus inhibiting the growth of fungi.

A recent study, developed by Omidi and Kakanejadifard [25], made modifications of chitosan and chitosan nanoparticles by long-chain pyridinium compounds via imine binding. In this work, the chitosan reaction was carried out in an acidic aqueous solution using the Bardoiu’s strategy, in which the yield of the Schiff base reaction was increased by the slow removal of water while the pyridinium salts were synthesized by the reaction between 4-pyridinecarboxaldehyde with N-alkyl bromides. The chitosan nanoparticles were prepared using a chitosan solution dissolved in acetic acid and added a TPP solution.The synthesis route of chitosan derivatives and nanoparticles is represented in Figure 6d. The bacterial activity of the synthesized derivatives against two Gram-positive bacteria *S. aureus* and *B. cereus* and two Gram-negative bacteria of *E. coli* and *K. pneumonia* were evaluated. The results induced that when introducing the quaternary pyridinium groups and forming the compounds CS10-CS16, one can observe an increase in the antibacterial properties against Gram-positive bacteria, however no noticeable improvement in the case of Gram-negative bacteria. The chitosan nanoparticles derivatives NP10-NP16 have greater antibacterial activities than the derivatives, where NP16 showed the greatest activity against an *S. aureus* bacterium with MIC = 81.25 μg/mL and MBC = 125 μg/mL. The results indicate that these derivatives may have purposes such as the synthesis of antimicrobial membranes, cosmetics, food and packaging industries.

In addition to the various N-pyridinium compounds that can be grafted into chitosan, some studies have shown the influence that N-pyridinium positions have on the properties of quaternized chitosan. Wei et al. [41], in their work, evaluated and synthesized double quaternary ammonium salts in distinct N-pyridinium positions and with analogous degrees of quaternization. The quaternary ammonium salts N-(2-pyridylmethyl), N-(3-pyridylmethyl) and N-(4-pyridylmethyl) were synthesized based on a reaction between the aldehyde group of carboxaldehyde pyridine and the primary amino group of chitosan followed by a reduction with sodium borohydride, the secondary amine and N-pyridine being attacked by iodomethane. From this, it was synthesized the double quaternized chitosans N-(1-methylpyridin-2-ylmethyl)-N,N-dimethyl chitosan, N-(1-methylpyridin-3-ylmethyl)-N,N-dimethyl chitosan and N-(1-methylpyridin-3-ylmethyl)-N, N-dimethyl chitosan (Figure 6e). It was observed that the derivative N-(1-methylpyridin-3-ylmethyl)- N,N-dimethyl chitosan showed a better oxidizing ability compared to other derivatives, this result was also observed by Li et al. [183] who realized that stronger electron groups have a better influence on antioxidant activity. This feature about the position of the N-pyridinium was also observed by Sajomsang et al. [31], where the authors investigated the influence of position on transfection efficiency and cytotoxicity in gene delivery. In this study, methylated N-pyridylmethyl chitosan chlorides (M-PyMeChCs) with a similar degree of total quaternization and molecular weight, but with N-pyridinium in different positions were synthesized by the reductive amination process.

## 5. Quaternized Chitosan with Phosphonium Salts

**Figure 7:**
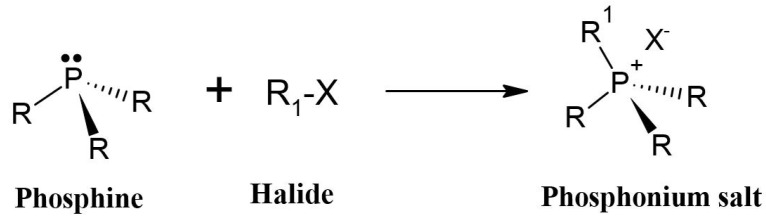
Nucleophilic substitution reaction between phosphine and halide.

Quaternary phosphonium salts are a multifunctional organophosphate class that are generally obtained by the nucleophilic substitution reaction between phosphine and halide [184], as shown in Figure 7. These salts have a structure that is similar to the salts of quarternary ammonium. However, they have better antimicrobial activity than ammonium salts [8]. According to Kenawy and Kandil [185], polymers with quaternary phosphonium groups are probably the most explored type of polymeric biocide. It is generally accepted that the mechanism of bactericidal action of polycationic biocides involves destructive interaction with the cell wall and/or cytoplasmic membranes.

**Figure 8:**
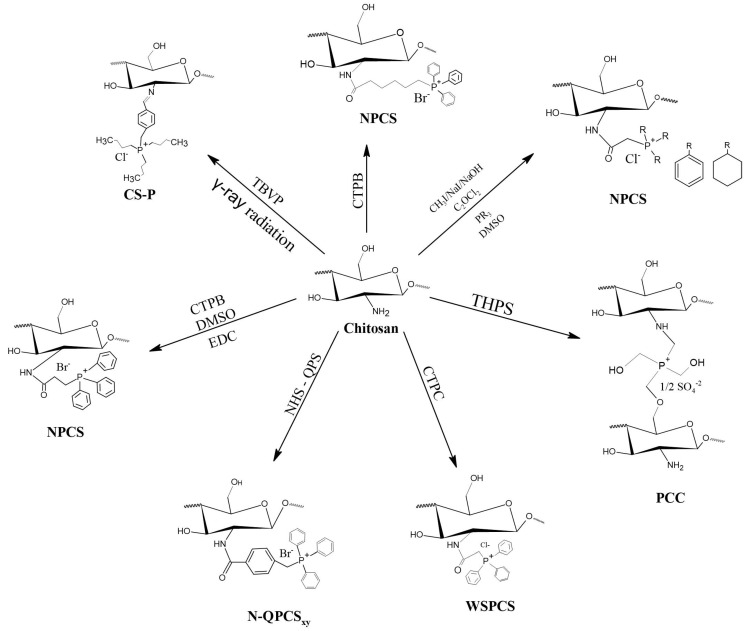
Synthetic route to obtain different quaternized chitosan derivatives with phosphonium salts.

Like pyridinium salts, the insertion of quaternary phosphonium salts into the chitosan structure is recent. Chitosan functionalization with phosphonium groups has been investigated for antibacterial applications [32,186,187,188], gene therapy, carrier of genes [27], vaccine antigen carrier [37] and adsorption in water treatment [42]. Figure 8 shows the simplified synthesis routes for quaternized chitosan compounds with phosphonium salts.

In 2011, Wang et al. [26] synthesized new chitosan derivatives with water-soluble quaternary phosphonium salts (WSPCSs) with different DQs (3.6% and 4.2%). The preparation and synthesis took place by stirring the chitosan with hydroxybenzotriazole in pure water at 25 °C until a homogeneous solution was obtained. The solution was mixed with CTPC followed by dropwise addition of EDC-HCl in ultra pure water and the reaction took place over a period of 48 h at 25 °C. The results presented by the authors showed a good solubility of WSPCSs in water without the addition of acid and an affinity for organic compounds, which can be dissolved in a solvent with a mixture of water and organic compounds. In addition, the authors also evaluated the cytotoxicity of these new derivatives for mouse fibroblasts, showing low toxicity with increasing the degree of substitution, due to the interactions between cationic polymers and plasma membrane components.

Qian et al. [40] synthesized new quaternized chitosan derivatives with (4-carboxybutyl)triphenyl phosphonium bromide (CTPB), using the same synthetic route presented by Wang et al. [26], with two different DQs (12.1% and 21.5%). These new derivatives, NPCSs, have been tested with several non-viral gene vectors. The authors observed that the insertion of the phosphonium group conferred greater cytotoxicity when compared to chitosan, since the introduction of the CTPB group ends up introducing positive charges to the structure of chitosan, a result also observed by Wang et al. [26], which was that positive charges interacted with the components of the cell’s plasma membrane. Continuing this work, Guo et al. [186] synthesized chitosan derivatives with phosphonium salts (NPCSs) with different DQs (3%, 13% and 21%) and tested the bacterial activity of these new derivatives against *E. coli* and two strains of *S. aureus*. The indispensable results show that, for lower degrees of substitution, the MIC and MBC values required by NPCS derivatives are lower when compared with derivatives such as HTCC, chitosan itself and CTPB for the three bacteria tested. The MIC and MBC values vary between 64 and 200 (μg/mL) and 200 to 400 (μg/mL), respectively. In order to apply NPCS with a new intramuscular vaccine antigen carrier, Cai et al. [37] proposed a change in the structure of the NPCS by quaternizing the chitosan with (2-carboxyethyl) triphenylphosphonium bromide. The synthesis route for this new derivative was the same as previously mentioned. Immunization tests performed using ovalbumin as the antigen models showed that immunization showed that the formulation of this new NPCS can contribute to a significantly higher level of antigen-specific immune responses, including a higher antigen-specific IgG antibody titer. With these results, the authors can state that this new water-soluble chitosan derivative can be used as a potential antigen carrier for prevention and immunization therapy.

*Via* trimethylation, chlorine acetylation and quaternization with tricyclohexylphosphine and triphenylphosphine, Tan et al. [32,188] synthesized two new chitosan derivatives modified with quaternary phosphonium salts: tricyclohexylphosphonium acetyl chitosan chloride (TCPACSC) and triphenylphosphonium acetyl chitosan chloride (TPPACSC). The derivatives were synthesized by a single chemical step, reacting chloroacetyl chitosan with tricyclohexylphosphine and triphenylphosphine. As shown by Wang et al. [26], the authors observed that the insertion of phosphonium salts in the chitosan structure improved its solubility, especially at alkaline pH. The authors evaluated the efficiency of these new derivatives in terms of their antifungal activities against the pathogens *P. asparagi*, *W. fusarium*, *C. lagenarium* and *F. oxysporum*, where the results indicated that even at concentrations below 0.5 μg/mL, the derivatives showed an inhibitory index higher than 70%. This high inhibitory effect is related to the polycations present in the structure of the phosphonium salts and according to Fan et al. [189], these polications end up adhering to the external membranes of the fungi, disturbing the surface of the microbial cell, preventing the transport of essential nutrients for cell survival in addition to causing the leakage of cellular constituents.

The application of these quaternized derivatives with phosphonium salts in wastewater treatment has been attracting interest in studies recently. Sessarego et al. [42] synthesized a new derivative from a single-step chemical reaction, where it incorporated phosphonium salts to chitosan, producing a phosphonium crosslinked chitosan (PCC). The preparation method was done by dispersing chitosan in a solution of acetic acid and adding tetrakis (hydroxymethyl) phosphonium sulfate. The PCC was evaluated for its Cr(VI) absorbing capacity in aqueous solutions and, although the results showed a greater absorption capacity of chitosan than the PCC, the absorption force shown by the PCC was greater due to the phosphonium molecule present in its structure to have a greater affinity with Cr(VI).

In addition to conventional methods for quaternizing chitosan with phosphonium salts, a new method has been proposed by Zeng et al. [27]. In this new method, the authors synthesized it by means of a graft copolymerization induced by γ radiation of poly(tributyl-(4-vinylbenzyl) phosphonium) in an acidic chitosan solution. They observed that the insertion of phosphonium salt in the chitosan structure gave it a better binding capacity between plasmids of enhanced green fluorescent proteins (pEGFP), and from an agarose gel electrophoresis assay and MTT assay, the authors were able to check that the CS-P particles loaded with pEGFP showed excellent biosafety. Tests carried out in vitro and in vivo with HeLa cells showed that CS-P loaded with pEGFP exhibits a high efficiency of gene transfection, making it a useful and viable way to obtain new gene vectors.

## 6. Other Quaternized Chitosan Derivatives

As the quaternization of chitosan has already demonstrated the possibility of obtaining materials with improved properties, many studies have expanded this modification using a number of reagents and obtaining a variety of quaternized chitosans, in addition to the most common ones aforementioned. Some of the options for obtaining different quaternized derivatives involve modifying the most common quaternized chitosans, such as TMC and HTCC, or using their precursors to obtain other quaternizing agents.

Xu et al. [20] developed compounds of N,O-di-quaternary ammonium chitosan with different degrees of O-substitution, by the reaction of TMC with CTA. All derivatives showed better bacterial activity than chitosan and N,N,N-trimethyl-O-(2-hydroxy-3-trimethylammonium propyl) chitosan, obtained after the O-quaternization step, demonstrated stronger activity than that of TMC, with an increase proportional to the degree of O-substitution. Qi et al. [190] synthesized the quaternary ammonium salt 3-chloro-2-hydroxypropyl dimethyl dehydroabietyl ammonium chloride (CHPDMDHA), from dehydroabietylamine, with a similar structure to that of CTA. CHPDMDHA, when reacting with low molecular weight chitosan (LWCS), generates a grafted polymeric cationic surfactant, called LWCS-g-CHPDMDHA. The properties of the surfactant were directly influenced by DQ: the critical micellar concentration (CMC), above which aggregates (or micelles) of surfactant begin to form, decreased with increasing DQ, as well as the surface tension in CMC.

According to Holappa et al. [191], the traditional route of obtaining TMC does not allow achieving structurally uniform polymers, since obtaining TMC does not occur without also methylating hydroxyl fractions of chitosan. To compensate, the authors suggested the synthesis of another quaternized derivative of chitosan, the chitosan N-betainates. Through subsequent steps of protection and deprotection of the amino and hydroxyl groups, it is obtained a full N-substituted chitosan, with a quaternary betaine moiety. The authors found a low antibacterial activity in neutral conditions and an increase in this activity with decreased DQ in acidic conditions. This means that the location of the positive charge in relation to the chitosan backbone affects antimicrobial activity. The same research group reported in Holappa et al. [192] obtaining another quaternized derivative of chitosan, in order to eliminate the problems of obtaining TMC: the mono- and di-quaternary piperazine derivatives. In Korjamo et al. [193], the research group evaluated the effect of these novel chitosan derivatives (N-betainates and N-piperazines) on the paracellular transport of mannitol and its cytotoxicity. The authors found an increase in paracellular transport in compounds with lower DQs and, in addition, N-betainates proved to be less toxic than N-piperazines, although both were found to have low toxicity. Once again, the authors proved that the permanent positive charge of the betaine and piperazine groups does not resemble the positive charge in the free amino group in terms of biological activity. Thus, the substitutes must be present at their minimum value until the pH-independent solubility is reached, in order to exercise its maximum activity.

Zambito et al. [194] and Zambito et al. [195] reported that the property of enhanced transmucosal absorption of drugs in common quaternized chitosans, such as TMC, is affected by molecular weight, DQ and structural features. Thus, the authors proposed novel quaternized chitosans instead of the traditional TMC to improve the absorption of intraocular drugs, generating the so-called N,O-[N,N-diethylaminomethyl (diethyldimethylene ammonium)n] methyl chitosans. The obtained derivatives enhanced the penetration of hydrophilic and hydrophobic molecules through the porcine buccal epithelium, with a remarkably stronger effect than that of TMC. Drug penetration through the paracellular route has been improved by all derivatives, while the transcellular route has been enhanced by derivatives with high DQ. Regarding the ex vivo and in vivo permeability through the cornea of a rabbit, all derivatives improved the permeability, with better results than the TMC and more effective for the highest DQ.

With the purpose of obtaining a quaternary chitosan derivative different from the traditional N-substituted ones, Cao et al. [196] synthesized N,N-dimethyl-O-quaternary ammonium chitosan using GTMAC as a quaternizing agent. The generated product had better moisture absorption and retention capacity than chitosan, in addition to stronger antibacterial activity. Li et al. [197] also used GTMAC to produce a novel O-quaternary ammonium N-acyl thiourea chitosan (OQCATUCS), containing double antimicrobial groups: the quaternary ammonium moiety and the thiourea group. An initial N-protection step was performed for the grafting of the quaternary ammonium group into the hydroxyl group, followed by the N-deprotection for reaction of the amino group with chloroacetylthiourea, generating an increase in the electropositivity charge of chitosan. For this reason, OQCATUCS showed better antimicrobial activity than O-quaternized chitosan and pure chitosan against *S. aureus*, *E. coli*, *A. niger*, *P. aeruginosa* and *B. subtillis*.

In Pan et al. [198], a reagent that is similar to GTMAC, but with a longer chain (epoxypropyl dodecyl dimethyl quaternary ammonium salt), was synthesized to act as a quaternizing agent. The N-quaternized compound was further reacted with N-methylolacrylamide to obtain O-acrylamidomethyl-N-[(2-hydroxy-3-dimethyl dodecyl ammonium) propyl] chitosan chloride (NMA-HDCC), with the purpose of application as a functional textile finishing. The addition of long chain alkyl groups to the chitosan backbone can improve membrane permeability, cell dissolution and antibacterial activity, in addition to allowing covalent bonding with tissue fibers. Cotton fabrics treated with NMA-HDCC demonstrated antibacterial characteristics, even after 30 repeated launderings. The action of NMA-HDCC was compared to chitosan, HTCC, HDCC and NMA-HTCC, and better activity was observed for chitosan derivatives, although only compounds with NMA showed a bacterial reduction of almost 100% and antibacterial activity after 30 launderings due to the covalent bonds of their vinyl groups with the fabrics. In addition, the HDCC compounds showed slightly better results than the HTCC series.

Cai et al. [199] modified HTCC by the esterification reaction in the hydroxyl group of chitosan, an alternative widely used to obtain better characteristics of solubility and antibacterial activity of chitosan. In general, chitosan ester is obtained by blocking the amino group followed by removal of the amino-protecting group. By esterifying HTCC, there is an amino-protected reagent and a dispensable removal step, as it is possible to take advantage of its valuable properties in the final product (O-acetyl-chitosan-N-2-hydroxypropyl trimethyl ammonium chloride—AQTS). The amphiphilic AQTS showed excellent solubility in organic solvents, water and aqueous solutions, however the increase in the degree of acetyl generated an increase in solubility in organic solvents and a decrease in aqueous solutions, since the acetyl groups provide greater hydrophobicity to the molecule. AQTS also showed better antibacterial activity than chitosan and HTCC, but the increase in the degree of acetyl was harmful for this property, since it generates compounds with less positive charge per unit mass, the main requirement for bacteria inhibition.

Zhang et al. [200] also developed an amphiphilic derivative of quaternary ammonium chitosan (2-N-carboxymethyl-6-O-diethylaminoethyl chitosan) by introducing carboxymethyl groups to the free amino groups of O-quaternized chitosan, with a view to enhance its solubility and enable its use in drug delivery. The developed compounds demonstrated very good blood compatibility and low cytotoxicity, and can be considered a safe material for the intended application. Using vitamin B12 (VB12) as a model drug, the authors obtained a controlled release system, possibly because the amphiphilic derivative allows to obtain microspheres with hydrophobic inner core and hydrophilic outer shell, which surround the hydrophilic molecule of VB12 and control its release. In the case of Pedro et al. [201], amphiphilic derivatives (BPTADDACH) were also produced with a view to application in drug delivery. However, both the quaternizing agent ((5-bromopentyl) trimethylammonium bromide) grafting and the step of adding dodecyl aldehyde as a hydrophobic chain occurred through the several chitosan amino groups. As a result, amphiphilic derivatives were able to self-assemble in aqueous solution to form aggregates, due to the different types of possible interactions (hydrogen bonds between hydrophilic segments or between hydrophilic segments and water molecules, and interactions between hydrophobic chains), without requiring the addition of another reagent. Moreover, they had the potential to carry hydrophobic drugs.

Another option is to quaternize previously modified chitosans. Han and Lin [202] quaternized N-(aryl) chitosans by introducing CTA in the hydroxyl groups of the chitosan chains, in two stages. Initially, chitosan reacted with different aromatic aldehydes (vanillin, cinnamaldehyde and benzaldehyde) to introduce an aromatic group in the amino group of chitosan via Schiff’s base. As a result, an extension of N-substitution of almost 100% was achieved, allowing the next stage, of CTA grafting, to occur predominantly by the hydroxyl groups of chitosan. Tests to evaluate the microbial activity of the obtained compounds showed that the quaternized N-aromatic chitosan compounds showed superior antibacterial action against Gram-negative and Gram-positive bacteria than unmodified chitosan, with the most effective action on Gram-negative bacteria rather than on Gram-positive. In addition, the compounds showed antifungal activity, which was not observed by unmodified chitosan. Comparing the three aromatic substituents, the authors found that the presence of hydroxyl phenol groups improves antibacterial activity, being even more effective than the positive charge density of quaternized chitosan.

Badawy and Rabea [203] also quaternized N-(aryl) chitosans containing different aromatic ligands (cinnamaldehyde, cuminaldehyde and 4-dimethylaminobenzaldehyde), however, through the chitosan amino group itself, obtaining compounds in the form N,N,N-(diethylaryl) chitosans chloride. The authors aimed to evaluate the in vitro and in vivo antifungal efficiency of these chitosan derivatives against the pathogenic fungus *Botrytis cinerea*, responsible for gray-mold disease, one of the diseases that most affects fruits and vegetables around the world. Quaternized derivatives showed greater antifungal inhibition than N-(aryl) chitosans and all of them showed results much higher than unmodified chitosan. In addition, in assays with tomato plant leaves, the obtained compounds were able to control the infection caused by *B. cinerea* and increase the resistance of plants to this disease. Sang et al. [204] quaternized the carboxymethyl chitosan (CMCH), a water soluble chitosan derivative with low antimicrobial activity, through the insertion of guanidine groups, a class of compounds known for its broad antimicrobial spectrum. The QGCMCHs indicated greater antimicrobial activity than chitosan over a wide pH range, and, when applied as food preservatives (in this case, strawberry preservatives), they demonstrated a better ability to prevent water loss and, therefore, prolong the shelf life.

Rahimi et al. [205] developed films of a novel quaternized chitosan and also of quaternized chitosan nanocomposites with silver nanoparticles (Ag NPs), aiming at its application as a wound dressing. The novel quaternized chitosans (QC-IMDZ) were obtained by grafting quaternary imidazole into the hydroxyls on the chitosan surface, through an intermediate N-protection step of the chitosan. All films, including those without AgNPs, showed high antimicrobial activity against different strains of bacteria and fungi, which was attributed to the presence of cationic imidazole. Regarding the properties suitable for application in wound dressings, the results showed that films with a greater amount of Ag NPs had a lower swelling ratio and less compatibility with blood, due to the increase in their hemolytic activity. In addition, when evaluating the blood’s clotting ability, films of pure QC-IMDZ demonstrated the best result. However, films with low amounts of Ag NPs contributed to reduced clotting time.

Table 1 shows the structural formulae for all compounds mentioned in this section, together with the applications or potential applications of these compounds.

## 7. Future Prospects

Quaternary chitosan derivatives have attracted a lot of attention in the last two decades because of the remarkable properties that these materials present. While quaternization preserves the excellent characteristics of chitosan, it broadens the range of possible applications as major limitations of the original polymer, such as poor solubility in neutral and alkaline media, are overcome. This way, quaternized chitosan is a multifunctional material that shows a lot of possible uses, showing potential especially in inactivation/inhibition of microorganisms, drug and gene delivery, vaccine adjuvancy, adsorption and wound dressing.

Even though the most important chitosan derivatives, such as TMC and HTCC, have been known for decades, research interest has only been intensified in the last few years. Still, their synthesis processes are not very well optimized and each method has always major drawbacks that hinder its use, either because of its toxicity or either because it is not economically feasible in industrial applications. Additionally, studies involving other quaternized chitosan compounds like phosphonium and pyridinium salts are very recent and still need maturation.

For the future, as there is growing interest in quaternized chitosan and the number of studies about it increases every year, it is expected that in the next years there will be enough knowledge about these compounds and that their preparation methods will be sufficiently optimized so that real applications can benefit from the formidable and diverse properties that these materials can offer.

## Figures and Tables

**Table 1:** Structural formulae of the other quaternized chitosan derivatives.

Quaternized Chitosan Derivatives	Structural Formula	Application/Potential Applications	Source
N,N,N-trimethyl O-(2-hydroxy-3- trimethylammonium propyl) chitosan	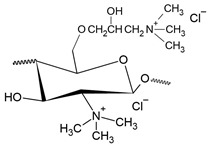	Antibacterial agent	[20]
LWCS-g-CHPDMDHA	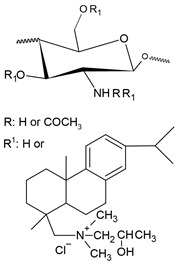	Polymeric surfactants for medicines, emulsions and treatment of wastewater	[190]
N-betainate chitosan	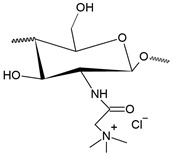	Antibacterial agent	[192]
N-piperazine chitosan	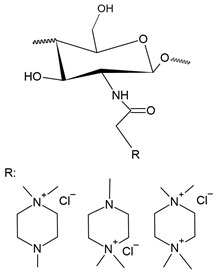	Pharmaceutical applications	[191]
N,O-[N,N-diethylaminomethyl (diethyldimethylene ammonium)${}_{n}$]methyl chitosans	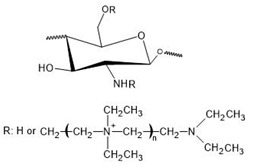	Intraocular drug delivery	[195]
N,N-dimethyl-O-quaternary ammonium chitosan	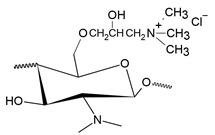	Moisturizer for cosmetics, biomedical materials, and antibacterial agents	[196]
OQCATUCS	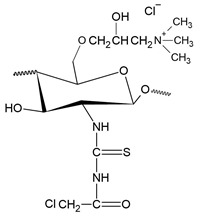	Bactericide in the fields of medicine and food	[197]
NMA-HDCC	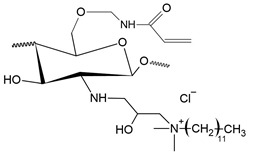	Functional finishing textiles	[198]
O-acetyl-chitosan- N-2-hydroxypropyl trimethyl ammonium chloride (C)	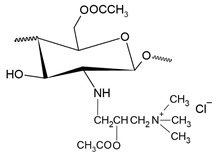	Food preservative	[199]
2-N-carboxymethyl-6-O -diethylaminoethyl chitosan	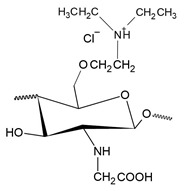	Drug delivery	[200]
BPTADDACH	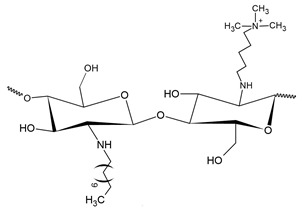	Drug delivery	[201]
Quaternized N-(aryl) chitosans	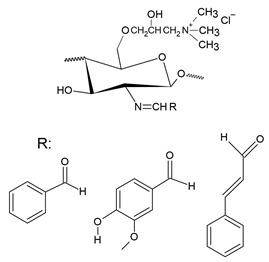	Antibacterial agent	[202]
N,N,N-(diethylaryl) chitosans chloride	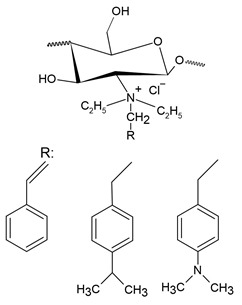	Fungicide	[203]
QGCMCH	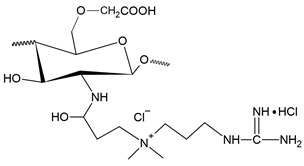	Food preservative	[204]
QC-IMDZ	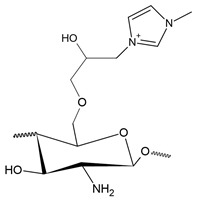	Wound dressings	[205]

## References

[1] Muñoz-Bonilla A., Cerrada M., Fernández-García M. (2014). Polymeric Materials with Antimicrobial Activity.

[2] Sharma S., Kumar A., Deepak, Kumar R., Rana N.K., Koch B. (2018). Development of a novel chitosan based biocompatible and self-healing hydrogel for controlled release of hydrophilic drug. Int. J. Biol. Macromol..

[3] Acevedo C.A., Olguín Y., Briceño M., Forero J.C., Osses N., Díaz-Calderón P., Jaques A., Ortiz R. (2019). Design of a biodegradable UV-irradiated gelatin-chitosan/nanocomposed membrane with osteogenic ability for application in bone regeneration. Mater. Sci. Eng. C.

[4] Anraku M., Gebicki J.M., Iohara D., Tomida H., Uekama K., Maruyama T., Hirayama F., Otagiri M. (2018). Antioxidant activities of chitosans and its derivatives in in vitro and in vivo studies. Carbohydr. Polym..

[5] Kumar D., Kumar P., Pandey J. (2018). Binary grafted chitosan film: Synthesis, characterization, antibacterial activity and prospects for food packaging. Int. J. Biol. Macromol..

[6] Kumar S., Deepak V., Kumari M., Dutta P.K. (2016). Antibacterial activity of diisocyanate-modified chitosan for biomedical applications. Int. J. Biol. Macromol..

[7] Zhang W., Dong P., Lao F., Liu J., Liao X., Wu J. (2019). Characterization of the major aroma-active compounds in Keitt mango juice: Comparison among fresh, pasteurization and high hydrostatic pressure processing juices. Food Chem..

[8] Qin Y., Li P., Guo Z. (2020). Cationic chitosan derivatives as potential antifungals: A review of structural optimization and applications. Carbohydr. Polym..

[9] Sajomsang W., Gonil P., Tantayanon S. (2009). Antibacterial activity of quaternary ammonium chitosan containing mono or disaccharide moieties: Preparation and characterization. Int. J. Biol. Macromol..

[10] Ignatova M., Manolova N., Rashkov I. (2007). Novel antibacterial fibers of quaternized chitosan and poly(vinyl pyrrolidone) prepared by electrospinning. Eur. Polym. J..

[11] Tan H., Ma R., Lin C., Liu Z., Tang T. (2013). Quaternized chitosan as an antimicrobial agent: Antimicrobial activity, mechanism of action and biomedical applications in orthopedics. Int. J. Mol. Sci..

[12] Curti E., de Britto D., Campana-Filho S.P. (2003). Methylation of Chitosan with Iodomethane: Effect of Reaction Conditions on Chemoselectivity and Degree of Substitution. Macromol. Biosci..

[13] Liu B., Shen S., Luo J., Wang X., Sun R. (2013). One-pot green synthesis and antimicrobial activity of exfoliated Ag NP-loaded quaternized chitosan/clay nanocomposites. RSC Adv..

[14] Rúnarsson Ö.V., Holappa J., Nevalainen T., Hjálmarsdóttir M., Järvinen T., Loftsson T., Einarsson J.M., Jónsdóttir S., Valdimarsdóttir M., Másson M. (2007). Antibacterial activity of methylated chitosan and chitooligomer derivatives: Synthesis and structure activity relationships. Eur. Polym. J..

[15] Hao J., Qin T., Zhang Y., Li Y., Zhang Y. (2019). Synthesis, surface properties and antimicrobial performance of novel gemini pyridinium surfactants. Colloids Surf. Biointerfaces.

[16] Rabea E.I., Badawy M.E., Stevens C.V., Smagghe G., Steurbaut W. (2003). Chitosan as antimicrobial agent: Applications and mode of action. Biomacromolecules.

[17] Fei Liu X., Lin Guan Y., Zhi Yang D., Li Z., De Yao K. (2001). Antibacterial action of chitosan and carboxymethylated chitosan. J. Appl. Polym. Sci..

[18] Rathinam S., Ólafsdóttir S., Jónsdóttir S., Hjálmarsdóttir M., Másson M. (2020). Selective synthesis of N,N,N-trimethylated chitosan derivatives at different degree of substitution and investigation of structure-activity relationship for activity against P. aeruginosa and MRSA. Int. J. Biol. Macromol..

[19] Jia Z., shen D., Xu W. (2001). Synthesis and antibacterial activities of quaternary ammonium salt of chitosan. Carbohydr. Res..

[20] Xu T., Xin M., Li M., Huang H., Zhou S., Liu J. (2011). Synthesis, characterization, and antibacterial activity of N,O-quaternary ammonium chitosan. Carbohydr. Res..

[21] Lim S.H., Hudson S.M. (2004). Synthesis and antimicrobial activity of a water-soluble chitosan derivative with a fiber-reactive group. Carbohydr. Res..

[22] Cho J., Grant J., Piquette-Miller M., Allen C. (2006). Synthesis and physicochemical and dynamic mechanical properties of a water-soluble chitosan derivative as a biomaterial. Biomacromolecules.

[23] Tan W., Li Q., Dong F., Zhang J., Luan F., Wei L., Chen Y., Guo Z. (2018). Novel cationic chitosan derivative bearing 1,2,3-triazolium and pyridinium: Synthesis, characterization, and antifungal property. Carbohydr. Polym..

[24] Jia R., Duan Y., Fang Q., Wang X., Huang J. (2016). Pyridine-grafted chitosan derivative as an antifungal agent. Food Chem..

[25] Omidi S., Kakanejadifard A. (2019). Modification of chitosan and chitosan nanoparticle by long chain pyridinium compounds: Synthesis, characterization, antibacterial, and antioxidant activities. Carbohydr. Polym..

[26] Wang L., Xu X., Guo S., Peng Z., Tang T. (2011). Novel water soluble phosphonium chitosan derivatives: Synthesis, characterization and cytotoxicity studies. Int. J. Biol. Macromol..

[27] Zeng K., Lin F.X., Xie J., Wang M.Z., Rong J.L., Zhao Y., You Y.Z., Asif A., Ge X.W. (2017). Chitosan modified by *γ*-ray-induced grafting of poly(tributyl-(4-vinylbenzyl)phosphonium) as a biosafe and high-efficiency gene carrier. New J. Chem..

[28] Chen H., Wang J.H., Liu C.D., Wang Y., Fu Y.N., Wang D., Sun H., Peng Y., Jiang M., Pu D.J. (2019). The effect of amphiphilic N,N,N-trimethyl-O-octadecyl chitosan on the oral bioavailability of acyclovir. J. Drug Deliv. Sci. Technol..

[29] Kim Y.H., Choi H.M., Yoon J.H. (1998). Synthesis of a Quaternary Ammonium Derivative of Chitosan and Its Application to a Cotton Antimicrobial Finish. Text. Res. J..

[30] Zhu Y., Pei H., Hu W., Jin Y., Xu H., Ren Y., Xue D. (2016). Effect of chitosan quaternary ammonium salt on the growth and microcystins release of: Microcystis aeruginosa. RSC Adv..

[31] Sajomsang W., Gonil P., Ruktanonchai U.R., Petchsangsai M., Opanasopit P., Puttipipatkhachorn S. (2014). Effect of N-pyridinium positions of quaternized chitosan on transfection efficiency in gene delivery system. Carbohydr. Polym..

[32] Tan W., Zhang J., Luan F., Wei L., Chen Y., Dong F., Li Q., Guo Z. (2017). Design, synthesis of novel chitosan derivatives bearing quaternary phosphonium salts and evaluation of antifungal activity. Int. J. Biol. Macromol..

[33] Lang G., Wendel H., Konrad E. (1990). Process for Making Quaternary Chitosan Derivatives for Cosmetic Agents. U.S. Patent.

[34] Mi F.L., Shyu S.S., Chen C.T., Lai J.Y. (2001). Adsorption of indomethacin onto chemically modified chitosan beads. Polymer.

[35] Kamiński K., Szczubiałka K., Zazakowny K., Lach R., Nowakowska M. (2010). Chitosan derivatives as novel potential heparin reversal agents. J. Med. Chem..

[36] Asasutjarit R., Theerachayanan T., Kewsuwan P., Veeranodha S., Fuongfuchat A., Ritthidej G.C. (2015). Development and Evaluation of Diclofenac Sodium Loaded-N-Trimethyl Chitosan Nanoparticles for Ophthalmic Use. AAPS PharmSciTech.

[37] Cai J., Zhang W., Xu J., Xue W., Liu Z. (2017). Evaluation of N-phosphonium chitosan as a novel vaccine carrier for intramuscular immunization. J. Biomater. Appl..

[38] Hecq J., Siepmann F., Siepmann J., Amighi K., Goole J. (2015). Development and evaluation of chitosan and chitosan derivative nanoparticles containing insulin for oral administration. Drug Dev. Ind. Pharm..

[39] de Britto D., Assis O.B.G. (2012). Aspectos químicos, bioquímicos e microbiológicos de sais quaternários de quitosana para revestimento ativo de maçãs fatiadas. Cienc. Tecnol. Aliment..

[40] Qian C., Xu X., Shen Y., Li Y., Guo S. (2013). Synthesis and preliminary cellular evaluation of phosphonium chitosan derivatives as novel non-viral vector. Carbohydr. Polym..

[41] Wei L., Li Q., Tan W., Dong F., Luan F., Guo Z., Saso L., Dux L., Wegrzyn G., Csont T. (2017). Synthesis, characterization, and the antioxidant activity of double quaternized chitosan derivatives. Molecules.

[42] Sessarego S., Rodrigues S.C., Xiao Y., Lu Q., Hill J.M. (2019). Phosphonium-enhanced chitosan for Cr(VI) adsorption in wastewater treatment. Carbohydr. Polym..

[43] Mohammad F., Arfin T., Al-Lohedan H.A. (2017). Enhanced biological activity and biosorption performance of trimethyl chitosan-loaded cerium oxide particles. J. Ind. Eng. Chem..

[44] Wu D., Zhu L., Li Y., Zhang X., Xu S., Yang G., Delair T. (2020). Chitosan-based Colloidal Polyelectrolyte Complexes for Drug Delivery: A Review. Carbohydr. Polym..

[45] Terayama H., Terayama E. (1948). High molecular antibacterial substances derived from chitin. About the manufacture of Macramin. J. Antibiot..

[46] Hatta S., Kuwabara S., Miyamoto H., Aoyama K., Utsunomiya N., Tanji S. (1950). Studies on macramin, a new high-molecular antibacterial substance derived from chitin. Jpn. Med J..

[47] Muzzarelli R.A., Tanfani F. (1985). The N-permethylation of chitosan and the preparation of N-trimethyl chitosan iodide. Carbohydr. Polym..

[48] Domard A., Rinaudo M., Terrassin C. (1986). New method for the quaternization of chitosan. Int. J. Biol. Macromol..

[49] le Dung P., Milas M., Rinaudo M., Desbrières J. (1994). Water soluble derivatives obtained by controlled chemical modifications of chitosan. Carbohydr. Polym..

[50] Polnok A., Borchard G., Verhoef J.C., Sarisuta N., Junginger H.E. (2004). Influence of methylation process on the degree of quaternization of N-trimethyl chitosan chloride. Eur. J. Pharm. Biopharm..

[51] Verheul R.J., Amidi M., van der Wal S., van Riet E., Jiskoot W., Hennink W.E. (2008). Synthesis, characterization and in vitro biological properties of O-methyl free N,N,N-trimethylated chitosan. Biomaterials.

[52] Sieval A.B., Thanou M., Kotzé A.F., Verhoef J.C., Brussee J., Junginger H.E. (1998). Preparation and NMR characterization of highly substituted N-trimethyl chitosan chloride. Carbohydr. Polym..

[53] Pardeshi C.V., Belgamwar V.S. (2016). Controlled synthesis of N,N,N-trimethyl chitosan for modulated bioadhesion and nasal membrane permeability. Int. J. Biol. Macromol..

[54] Sandri G., Rossi S., Bonferoni M.C., Ferrari F., Zambito Y., Di Colo G., Caramella C. (2005). Buccal penetration enhancement properties of N-trimethyl chitosan: Influence of quaternization degree on absorption of a high molecular weight molecule. Int. J. Pharm..

[55] Snyman D., Hamman J.H., Kotze A.F. (2003). Evaluation of the mucoadhesive properties of N-trimethyl chitosan chloride. Drug Dev. Ind. Pharm..

[56] Jintapattanakit A., Mao S., Kissel T., Junyaprasert V.B. (2008). Physicochemical properties and biocompatibility of N-trimethyl chitosan: Effect of quaternization and dimethylation. Eur. J. Pharm. Biopharm..

[57] Van der Lubben I.M., Verhoef J.C., Borchard G., Junginger H.E. (2001). Chitosan and its derivatives in mucosal drug and vaccine delivery. Eur. J. Pharm..

[58] Thanou M.M., Kotzé A.F., Scharringhausen T., Lueßen H.L., De Boer A.G., Verhoef J.C., Junginger H.E. (2000). Effect of degree of quaternization of N-trimethyl chitosan chloride for enhanced transport of hydrophilic compounds across intestinal Caco-2 cell monolayers. J. Control. Release.

[59] Florea B.I., Thanou M., Junginger H.E., Borchard G. (2006). Enhancement of bronchial octreotide absorption by chitosan and N-trimethyl chitosan shows linear in vitro/in vivo correlation. J. Control. Release.

[60] de Britto D., Assis O.B. (2007). A novel method for obtaining a quaternary salt of chitosan. Carbohydr. Polym..

[61] Sudarshan N.R., Hoover D.G., Knorr D. (1992). Antibacterial Action of Chitosan. Food Biotechnol..

[62] Vidar Rúnarsson Ö., Holappa J., Malainer C., Steinsson H., Hjálmarsdóttir M., Nevalainen T., Másson M. (2010). Antibacterial activity of N-quaternary chitosan derivatives: Synthesis, characterization and structure activity relationship (SAR) investigations. Eur. Polym. J..

[63] Sajomsang W., Ruktanonchai U.R., Gonil P., Warin C. (2010). Quaternization of N-(3-pyridylmethyl) chitosan derivatives: Effects of the degree of quaternization, molecular weight and ratio of N-methylpyridinium and N,N,N-trimethyl ammonium moieties on bactericidal activity. Carbohydr. Polym..

[64] Geng X., Yang R., Huang J., Zhang X., Wang X. (2013). Evaluation Antibacterial Activity of Quaternary-Based Chitin/Chitosan Derivatives In Vitro. J. Food Sci..

[65] Kim C.H., Choi J.W., Chun H.J., Choi K.S. (1997). Synthesis of chitosan derivatives with quaternary ammonium salt and their antibacterial activity. Polym. Bull..

[66] Belalia R., Grelier S., Benaissa M., Coma V. (2008). New Bioactive Biomaterials Based on Quaternized Chitosan. J. Agric. Food Chem..

[67] Sadeghi A.M., Dorkoosh F.A., Avadi M.R., Saadat P., Rafiee-Tehrani M., Junginger H.E. (2008). Preparation, characterization and antibacterial activities of chitosan, N-trimethyl chitosan (TMC) and N-diethylmethyl chitosan (DEMC) nanoparticles loaded with insulin using both the ionotropic gelation and polyelectrolyte complexation methods. Int. J. Pharm..

[68] De Britto D., Campana-Filho S.P. (2004). A kinetic study on the thermal degradation of N,N,N-trimethylchitosan. Polym. Degrad. Stab..

[69] Wu M., Long Z., Xiao H., Dong C. (2016). Recent research progress on preparation and application of N,N,N-trimethyl chitosan. Carbohydr. Res..

[70] Kulkarni A.D., Patel H.M., Surana S.J., Vanjari Y.H., Belgamwar V.S., Pardeshi C.V. (2017). N,N,N-Trimethyl chitosan: An advanced polymer with myriad of opportunities in nanomedicine. Carbohydr. Polym..

[71] Mao Z., Ma L., Yan J., Yan M., Gao C., Shen J. (2007). The gene transfection efficiency of thermoresponsive N,N,N-trimethyl chitosan chloride-g-poly(N-isopropylacrylamide) copolymer. Biomaterials.

[72] Zhang J., Tan W., Wang G., Yin X., Li Q., Dong F., Guo Z. (2018). Synthesis, characterization, and the antioxidant activity of N,N,N-trimethyl chitosan salts. Int. J. Biol. Macromol..

[73] Rúnarsson Ö.V., Malainer C., Holappa J., Sigurdsson S.T., Másson M. (2008). tert-Butyldimethylsilyl O-protected chitosan and chitooligosaccharides: Useful precursors for N-modifications in common organic solvents. Carbohydr. Res..

[74] Benediktsdóttir B.E., Gaware V.S., Rúnarsson Ö.V., Jónsdóttir S., Jensen K.J., Másson M. (2011). Synthesis of N,N,N-trimethyl chitosan homopolymer and highly substituted N-alkyl-N,N-dimethyl chitosan derivatives with the aid of di-tert- butyldimethylsilyl chitosan. Carbohydr. Polym..

[75] Wu M., Long Z., Xiao H., Dong C. (2017). Preparation of N, N, N-trimethyl chitosan via a novel approach using dimethyl carbonate. Carbohydr. Polym..

[76] Mahajan T., Bangde P., Dandekar P., Jain R. (2020). Greener approach for synthesis of N,N,N-trimethyl chitosan (TMC) using ternary deep eutectic solvents (TDESs). Carbohydr. Res..

[77] Mourya V.K., Inamdar N.N. (2009). Trimethyl chitosan and its applications in drug delivery. J. Mater. Sci. Mater. Med..

[78] Zhao X., Lu C., Yang S., Zhang J. (2020). Bioconjugation of aptamer to fluorescent trimethyl chitosan nanoparticles for bacterial detection. Mater. Lett..

[79] Sahni J.K., Chopra S., Ahmad F.J., Khar R.K. (2008). Potential prospects of chitosan derivative trimethyl chitosan chloride (TMC) as a polymeric absorption enhancer: Synthesis, characterization and applications. J. Pharm. Pharmacol..

[80] Kotzé A.F., De Leeuw B.J., Lueßen H.L., De Boer A.G., Verhoef J.C., Junginger H.E. (1997). Chitosans for enhanced delivery of therapeutic peptides across intestinal epithelia: In vitro evaluation in Caco-2 cell monolayers. Int. J. Pharm..

[81] He W., Guo X., Zhang M. (2008). Transdermal permeation enhancement of N-trimethyl chitosan for testosterone. Int. J. Pharm..

[82] Jonker C., Hamman J.H., Kotzé A.F. (2002). Intestinal paracellular permeation enhancement with quaternised chitosan: In situ and in vitro evaluation. Int. J. Pharm..

[83] Hamman J.H., Stander M., Kotzé A.F. (2002). Effect of the degree of quaternisation of N-trimethyl chitosan chloride on absorption enhancement: In vivo evaluation in rat nasal epithelia. Int. J. Pharm..

[84] Di Colo G., Burgalassi S., Zambito Y., Monti D., Chetoni P. (2004). Effects of different N-trimethyl chitosans on in vitro/in vivo ofloxacin transcorneal permeation. J. Pharm. Sci..

[85] Benediktsdóttir B.E., Gudjónsson T., Baldursson Ó., Másson M. (2014). N-alkylation of highly quaternized chitosan derivatives affects the paracellular permeation enhancement in bronchial epithelia in vitro. Eur. J. Pharm. Biopharm..

[86] Liu M., Zhang J., Zhu X., Shan W., Li L., Zhong J., Zhang Z., Huang Y. (2016). Efficient mucus permeation and tight junction opening by dissociable “mucus-inert” agent coated trimethyl chitosan nanoparticles for oral insulin delivery. J. Control. Release.

[87] Tsai L.C., Chen C.H., Lin C.W., Ho Y.C., Mi F.L. (2019). Development of mutlifunctional nanoparticles self-assembled from trimethyl chitosan and fucoidan for enhanced oral delivery of insulin. Int. J. Biol. Macromol..

[88] Ramalingam P., Ko Y.T. (2016). Improved oral delivery of resveratrol from N-trimethyl chitosan-g-palmitic acid surface-modified solid lipid nanoparticles. Colloids Surf. B Biointerfaces.

[89] Du Q., Chen J., Yan G., Lyu F., Huang J., Ren J., Di L. (2019). Comparison of different aliphatic acid grafted N-trimethyl chitosan surface-modified nanostructured lipid carriers for improved oral kaempferol delivery. Int. J. Pharm..

[90] He R., Yin C. (2017). Trimethyl chitosan based conjugates for oral and intravenous delivery of paclitaxel. Acta Biomater..

[91] Chen G., Svirskis D., Lu W., Ying M., Huang Y., Wen J. (2018). N-trimethyl chitosan nanoparticles and CSKSSDYQC peptide: N-trimethyl chitosan conjugates enhance the oral bioavailability of gemcitabine to treat breast cancer. J. Control. Release.

[92] Kontogiannidou E., Meikopoulos T., Virgiliou C., Bouropoulos N., Gika H., Vizirianakis I.S., Müllertz A., Fatouros D.G. (2020). Towards the development of Self-Nano-Emulsifying Drug Delivery Systems (SNEDDS) containing trimethyl chitosan for the oral delivery of amphotericin B: In vitro assessment and cytocompatibility studies. J. Drug Deliv. Sci. Technol..

[93] Pardeshi C.V., Belgamwar V.S. (2018). N,N,N‑trimethyl chitosan modified flaxseed oil based mucoadhesive neuronanoemulsions for direct nose to brain drug delivery. Int. J. Biol. Macromol..

[94] Pardeshi C.V., Agnihotri V.V., Patil K.Y., Pardeshi S.R., Surana S.J. (2020). Mannose-anchored N,N,N-trimethyl chitosan nanoparticles for pulmonary administration of etofylline. Int. J. Biol. Macromol..

[95] Li J., Jin X., Yang Y., Zhang L., Liu R., Li Z. (2020). Trimethyl chitosan nanoparticles for ocular baicalein delivery: Preparation, optimization, in vitro evaluation, in vivo pharmacokinetic study and molecular dynamics simulation. Int. J. Biol. Macromol..

[96] Rahmani S., Hakimi S., Esmaeily A., Samadi F.Y., Mortazavian E., Nazari M., Mohammadi Z., Tehrani N.R., Tehrani M.R. (2019). Novel chitosan based nanoparticles as gene delivery systems to cancerous and noncancerous cells. Int. J. Pharm..

[97] Baghaei M., Tekie F.S.M., Khoshayand M.R., Varshochian R., Hajiramezanali M., Kachousangi M.J., Dinarvand R., Atyabi F. (2021). Optimization of chitosan-based polyelectrolyte nanoparticles for gene delivery, using design of experiment: in vitro and in vivo study. Mater. Sci. Eng. C.

[98] Slütter B., Plapied L., Fievez V., Alonso Sande M., des Rieux A., Schneider Y.J., Van Riet E., Jiskoot W., Préat V. (2009). Mechanistic study of the adjuvant effect of biodegradable nanoparticles in mucosal vaccination. J. Control. Release.

[99] Hagenaars N., Verheul R.J., Mooren I., de Jong P.H., Mastrobattista E., Glansbeek H.L., Heldens J.G., van den Bosch H., Hennink W.E., Jiskoot W. (2009). Relationship between structure and adjuvanticity of N,N,N-trimethyl chitosan (TMC) structural variants in a nasal influenza vaccine. J. Control. Release.

[100] Verheul R.J., Hagenaars N., Van Es T., Van Gaal E.V., De Jong P.H., Bruijns S., Mastrobattista E., Slütter B., Que I., Heldens J.G. (2012). A step-by-step approach to study the influence of N-acetylation on the adjuvanticity of N,N,N-trimethyl chitosan (TMC) in an intranasal nanoparticulate influenza virus vaccine. Eur. J. Pharm. Sci..

[101] Sayin B., Somavarapu S., Li X.W., Sesardic D., Şenel S., Alpar O.H. (2009). TMC-MCC (N-trimethyl chitosan-mono-N-carboxymethyl chitosan) nanocomplexes for mucosal delivery of vaccines. Eur. J. Pharm. Sci..

[102] Cevher E., Salomon S.K., Somavarapu S., Brocchini S., Alpar H.O. (2015). Development of chitosan–pullulan composite nanoparticles for nasal delivery of vaccines: in vivo studies. J. Microencapsul..

[103] Nevagi R.J., Khalil Z.G., Hussein W.M., Powell J., Batzloff M.R., Capon R.J., Good M.F., Skwarczynski M., Toth I. (2018). Polyglutamic acid-trimethyl chitosan-based intranasal peptide nano-vaccine induces potent immune responses against group A streptococcus. Acta Biomater..

[104] Dabaghian M., Latifi A.M., Tebianian M., NajmiNejad H., Ebrahimi S.M. (2018). Nasal vaccination with r4M2e.HSP70c antigen encapsulated into N-trimethyl chitosan (TMC) nanoparticulate systems: Preparation and immunogenicity in a mouse model. Vaccine.

[105] Bal S.M., Slütter B., Verheul R., Bouwstra J.A., Jiskoot W. (2012). Adjuvanted, antigen loaded N-trimethyl chitosan nanoparticles for nasal and intradermal vaccination: Adjuvant- and site-dependent immunogenicity in mice. Eur. J. Pharm. Sci..

[106] Schipper P., van der Maaden K., Groeneveld V., Ruigrok M., Romeijn S., Uleman S., Oomens C., Kersten G., Jiskoot W., Bouwstra J. (2017). Diphtheria toxoid and N-trimethyl chitosan layer-by-layer coated pH-sensitive microneedles induce potent immune responses upon dermal vaccination in mice. J. Control. Release.

[107] Abkar M., Fasihi-Ramandi M., Kooshki H., Lotfi A.S. (2018). Intraperitoneal immunization with Urease loaded N-trimethyl Chitosan nanoparticles elicits high protection against Brucella melitensis and Brucella abortus infections. Immunol. Lett..

[108] Zhou Z., Yan D., Cheng X., Kong M., Liu Y., Feng C., Chen X. (2016). Biomaterials based on N,N,N-trimethyl chitosan fibers in wound dressing applications. Int. J. Biol. Macromol..

[109] Patrulea V., Laurent-Applegate L.A., Ostafe V., Borchard G., Jordan O. (2019). Polyelectrolyte nanocomplexes based on chitosan derivatives for wound healing application. Eur. J. Pharm. Biopharm..

[110] Romero R., Chubb L., Travers J.K., Gonzales T.R., Ehrhart N.P., Kipper M.J. (2015). Coating cortical bone allografts with periosteum-mimetic scaffolds made of chitosan, trimethyl chitosan, and heparin. Carbohydr. Polym..

[111] Tabriz A., Ur Rehman Alvi M.A., Khan Niazi M.B., Batool M., Bhatti M.F., Khan A.L.A.U., Khan A.L.A.U., Jamil T., Ahmad N.M. (2019). Quaternized trimethyl functionalized chitosan based antifungal membranes for drinking water treatment. Carbohydr. Polym..

[112] Bigogno R.G., Rodríguez R.J.S., Abreu M.d.F. (2018). Quaternized Chitosan for Ecological Treatment of Bauxite Mining Effluents. J. Polym. Environ..

[113] Abu Elella M.H., ElHafeez E.A., Goda E.S., Lee S., Yoon K.R. (2019). Smart bactericidal filter containing biodegradable polymers for crystal violet dye adsorption. Cellulose.

[114] Lang G., Konrad E., Wendel H., Muzzarelli R., Jeuniaux C., Gooday G.W. (1986). Chitosan derivatives: water-soluble products by reaction with epoxides. Proceedings of the Third International Conference on Chitin and Chitosan.

[115] Loubaki E., Ourevitch M., Sicsic S., Dunant R.H. (1991). Chemical Modification of Chitosan By Glycidyl Trimethylammonium chloride. Eur. Polym. J..

[116] Stefan J., Lorkowska-Zawicka B., Kaminski K., Szczubialka K., Nowakowska M., Korbut R. (2014). The current view on biological potency of cationically modified chitosan. J. Physiol. Pharmacol..

[117] Seong H.S., Whang H.S., Ko S.W. (2000). Synthesis of a Quaternary Ammonium Derivative of Chito-oligosaccharide as Antimicrobial Agent for Cellulosic Fibers. J. Appl. Polym. Sci..

[118] Yang X., Zhang C., Qiao C., Mu X., Li T., Xu J., Shi L., Zhang D. (2015). A simple and convenient method to synthesize N-[(2-hydroxyl)-propyl-3-trimethylammonium] chitosan chloride in an ionic liquid. Carbohydr. Polym..

[119] Jiang T., James R., Kumbar S.G., Laurencin C.T. (2014). Chitosan as a Biomaterial: Structure, Properties, and Applications in Tissue Engineering and Drug Delivery.

[120] Chen Y., Li J., Li Q., Shen Y., Ge Z., Zhang W., Chen S. (2016). Enhanced water-solubility, antibacterial activity and biocompatibility upon introducing sulfobetaine and quaternary ammonium to chitosan. Carbohydr. Polym..

[121] Shagdarova B., Lunkov A., Il’ina A., Varlamov V. (2019). Investigation of the properties of N-[(2-hydroxy-3-trimethylammonium) propyl] chloride chitosan derivatives. Int. J. Biol. Macromol..

[122] Wang L., Qin C., Wang W., Li W. (2011). Effect of orally administered N-(2-hydroxyl) propyl-3-trimethyl ammonium chitosan on the levels of iron, zinc, copper, calcium and lead in mice. Carbohydr. Polym..

[123] Xiao B., Wan Y., Wang X., Zha Q., Liu H., Qiu Z., Zhang S. (2012). Synthesis and characterization of N-(2-hydroxy)propyl-3-trimethyl ammonium chitosan chloride for potential application in gene delivery. Colloids Surf. B Biointerfaces.

[124] Huang J., Cheng Z.H., Xie H.H., Gong J.Y., Lou J., Ge Q., Wang Y.J., Wu Y.F., Liu S.W., Sun P.L. (2014). Effect of quaternization degree on physiochemical and biological activities of chitosan from squid pens. Int. J. Biol. Macromol..

[125] Zhou Y., Yang H., Liu X., Mao J., Gu S., Xu W. (2013). Potential of quaternization-functionalized chitosan fiber for wound dressing. Int. J. Biol. Macromol..

[126] Wang B., Yang X., Qiao C., Li Y., Li T., Xu C. (2018). Effects of chitosan quaternary ammonium salt on the physicochemical properties of sodium carboxymethyl cellulose-based films. Carbohydr. Polym..

[127] Wang Y.Q., Fan Q.Z., Liu Y., Yue H., Ma X.W., Wu J., Ma G.H., Su Z.G. (2016). Improving adjuvanticity of quaternized chitosan–based microgels for H5N1 split vaccine by tailoring the particle properties to achieve antigen dose sparing effect. Int. J. Pharm..

[128] Kim Y.H., Nam C.W., Choi J.W., Jang J. (2003). Durable antimicrobial treatment of cotton fabrics using N-(2-hydroxy)propyl-3-trimethylammonium chitosan chloride and polycarboxylic acids. J. Appl. Polym. Sci..

[129] Chi W., Qin C., Zeng L., Li W., Wang W. (2007). Microbiocidal activity of chitosan-N-2-hydroxypropyl trimethyl ammonium chloride. J. Appl. Polym. Sci..

[130] Qin C., Xiao Q., Li H., Fang M., Liu Y., Chen X., Li Q. (2004). Calorimetric studies of the action of chitosan-N-2-hydroxypropyl trimethyl ammonium chloride on the growth of microorganisms. Int. J. Biol. Macromol..

[131] Hoque J., Adhikary U., Yadav V., Samaddar S., Konai M.M., Prakash R.G., Paramanandham K., Shome B.R., Sanyal K., Haldar J. (2016). Chitosan Derivatives Active against Multidrug-Resistant Bacteria and Pathogenic Fungi: In Vivo Evaluation as Topical Antimicrobials. Mol. Pharm..

[132] Milewska A., Ciejka J., Kaminski K., Karewicz A., Bielska D., Zeglen S., Karolak W., Nowakowska M., Potempa J., Bosch B.J. (2013). Novel polymeric inhibitors of HCoV-NL63. Antivir. Res..

[133] Milewska A., Kaminski K., Ciejka J., Kosowicz K., Zeglen S., Wojarski J., Nowakowska M., Szczubiałka K., Pyrc K. (2016). HTCC: Broad range inhibitor of coronavirus entry. PLoS ONE.

[134] Milewska A., Chi Y., Szczepanski A., Barreto-Duran E., Liu K., Liu D., Guo X., Ge Y., Li J., Cui L. (2020). HTCC as a highly effective polymeric inhibitor of SARS-CoV-2 and MERS-CoV. bioRxiv.

[135] Lee E.J., Kim Y.H. (2010). Synthesis and thermo-responsive properties of chitosan-g-poly (N-isopropylacrylamide) and HTCC-g-poly(N-isopropylacrylamide) copolymers. Fibers Polym..

[136] Wu H., Zhang J., Xiao B., Zan X., Gao J., Wan Y. (2011). N-(2-hydroxypropyl)-3-trimethylammonium chitosan-poly(*ϵ*-caprolactone) copolymers and their antibacterial activity. Carbohydr. Polym..

[137] Mivehi L., Bahrami S.H., Malek R.M.A. (2008). Properties of Polyacrylonitrile-N-(2-hydroxy) propyl-3-trimethylammonium Chitosan Chloride Blend Films and Fibers. J. Appl. Polym. Sci..

[138] Li S.D., Li P.W., Yang Z.M., Peng Z., Quan W.Y., Yang X.H., Yang L., Dong J.J. (2014). Synthesis and characterization of chitosan quaternary ammonium salt and its application as drug carrier for ribavirin. Drug Deliv..

[139] Spinelli V.A., Laranjeira M.C., Fávere V.T. (2004). Preparation and characterization of quaternary chitosan salt: Adsorption equilibrium of chromium(VI) ion. React. Funct. Polym..

[140] Lu Y., Cheng D., Lu S., Huang F., Li G. (2014). Preparation of quaternary ammonium salt of chitosan nanoparticles and their textile properties on Antheraea pernyi silk modification. Text. Res. J..

[141] Wu Y., Wu S., Hou L., Wei W., Zhou M., Su Z., Wu J., Chen W., Ma G. (2012). Novel thermal-sensitive hydrogel enhances both humoral and cell-mediated immune responses by intranasal vaccine delivery. Eur. J. Pharm. Biopharm..

[142] Nam C.W., Kim Y.H., Ko S.W. (1999). Modification of polyacrylonitrile (PAN) fiber by blending with N-(2-hydroxy)propyl-3-trimethyl-ammonium chitosan chloride. J. Appl. Polym. Sci..

[143] Ruihua H., Bingchao Y., Zheng D., Wang B. (2012). Preparation and characterization of a quaternized chitosan. J. Mater. Sci..

[144] Song H., Wu H., Li S.J., Tian H., Li Y.R., Wang J.G. (2018). Homogeneous synthesis of cationic chitosan via new avenue. Molecules.

[145] Li W., Duan Y., Huang J., Zheng Q. (2016). Synthesis, antioxidant and cathepsin D inhibition activity of quaternary ammonium chitosan derivatives. Carbohydr. Polym..

[146] Zhang X., Geng X., Jiang H., Li J., Huang J. (2012). Synthesis and characteristics of chitin and chitosan with the (2-hydroxy-3-trimethylammonium)propyl functionality, and evaluation of their antioxidant activity in vitro. Carbohydr. Polym..

[147] Tao W., Zheng H.Q., Fu T., He Z.J., Hong Y. (2017). N-(2-hydroxy) propyl-3-trimethylammonium chitosan chloride: An immune-enhancing adjuvant for hepatitis E virus recombinant polypeptide vaccine in mice. Hum. Vaccines Immunother..

[148] Ali S.A., Singh R.P. (2009). Synthesis and characterization of a modified chitosan. Macromol. Symp..

[149] Tan Y., Wu H., Xie T., Chen L., Hu S., Tian H., Wang Y., Wang J. (2020). Characterization and antibacterial effect of quaternized chitosan anchored cellulose beads. Int. J. Biol. Macromol..

[150] Sun Y., Wan A. (2007). Preparation of Nanoparticles Composed of Chitosan and Its Derivatives as Delivery Systems for Macromolecules Yan. J. Appl. Polym. Sci..

[151] Soares P.I., Sousa A.I., Silva J.C., Ferreira I.M., Novo C.M., Borges J.P. (2016). Chitosan-based nanoparticles as drug delivery systems for doxorubicin: Optimization and modelling. Carbohydr. Polym..

[152] Wang F., Yao J., Russel M., Chen H., Chen K., Zhou Y., Ceccanti B., Zaray G., Choi M.M. (2010). Development and analytical application of a glucose biosensor based on glucose oxidase/O-(2-hydroxyl)propyl-3-trimethylammonium chitosan chloride nanoparticle-immobilized onion inner epidermis. Biosens. Bioelectron..

[153] Zhang S., Huang S., Lu L., Song X., Li P., Wang F. (2018). Curdlan sulfate-O-linked quaternized chitosan nanoparticles: Potential adjuvants to improve the immunogenicity of exogenous antigens via intranasal vaccination. Int. J. Nanomed..

[154] Cheah W.Y., Show P.L., Ng I.S., Lin G.Y., Chiu C.Y., Chang Y.K. (2019). Antibacterial activity of quaternized chitosan modified nanofiber membrane. Int. J. Biol. Macromol..

[155] Guang W.Y. (2003). The Effect of Chitosan and Its Derivatives on the Dyeability of Silk. Ph.D. Thesis.

[156] Gruškiene R., Deveikyte R., Makuška R. (2013). Quaternization of chitosan and partial destruction of the quaternized derivatives making them suitable for electrospinning. Chemija.

[157] Wan A., Xu Q., Sun Y., Li H. (2013). Antioxidant activity of high molecular weight chitosan and N,O-quaternized chitosans. J. Agric. Food Chem..

[158] Jin Y., Pei H., Hu W., Zhu Y., Xu H., Ma C., Sun J., Li H. (2017). A promising application of chitosan quaternary ammonium salt to removal of Microcystis aeruginosa cells from drinking water. Sci. Total. Environ..

[159] Vanitha P.D., Pandima D.M.K., Arumugam P., Sudharsan K., Anruradha V. (2017). Larvicidal Activities of N-(2-Hydroxyl) Propyl-3-Trimethyl Ammonium Chitosan Chloride (HTCC) and Silver Nanoparticles against Two Mosquito Species, Aedes and Culex: A Comparative Study. Res. Rev. J. Zool. Sci..

[160] Mi F.L., Shyu S.S., Chen C.T., Schoung J.Y. (1999). Porous chitosan microsphere for controlling the antigen release of Newcastle disease vaccine: Preparation of antigen-adsorbed microsphere and in vitro release. Biomaterials.

[161] Xu Y., Du Y., Huang R., Gao L. (2003). Preparation and modification of N-(2-hydroxyl) propyl-3-trimethyl ammonium chitosan chloride nanoparticle as a protein carrier. Biomaterials.

[162] Zhao S.H., Wu X.T., Guo W.C., Du Y.M., Yu L., Tang J. (2010). N-(2-hydroxyl) propyl-3-trimethyl ammonium chitosan chloride nanoparticle as a novel delivery system for Parathyroid Hormone-Related Protein 1-34. Int. J. Pharm..

[163] Lorkowska-Zawicka B., Kamiński K., Ciejka J., Szczubiałka K., Białas M., Okoń K., Adamek D., Nowakowska M., Jawień J., Olszanecki R. (2014). Inactivation of heparin by cationically modified chitosan. Mar. Drugs.

[164] Rosa S., Laranjeira M.C., Riela H.G., Fávere V.T. (2008). Cross-linked quaternary chitosan as an adsorbent for the removal of the reactive dye from aqueous solutions. J. Hazard. Mater..

[165] Zhang W., Sun C., Zhao Y., Lu X. (2011). One-pot synthesis and characterization of cross-linked quaternized chitosan microspheres as protein adsorbent. Int. J. Biol. Macromol..

[166] Ciejka J., Wolski K., Nowakowska M., Pyrc K., Szczubiałka K. (2017). Biopolymeric nano/microspheres for selective and reversible adsorption of coronaviruses. Mater. Sci. Eng. C.

[167] Li H., Du Y., Wu X., Zhan H. (2004). Effect of molecular weight and degree of substitution of quaternary chitosan on its adsorption and flocculation properties for potential retention-aids in alkaline papermaking. Colloids Surf. A Physicochem. Eng. Asp..

[168] Li H., Du Y., Xu Y., Zhan H., Kennedy J.F. (2004). Interactions of cationized chitosan with components in a chemical pulp suspension. Carbohydr. Polym..

[169] Wan Y., Peppley B., Creber K.A., Bui V.T., Halliop E. (2008). Quaternized-chitosan membranes for possible applications in alkaline fuel cells. J. Power Sources.

[170] An Y., Jiang G., Ren Y., Zhang L., Qi Y., Ge Q. (2015). An environmental friendly and biodegradable shale inhibitor based on chitosan quaternary ammonium salt. J. Pet. Sci. Eng..

[171] Grant J., Lee H., Soo P.L., Cho J., Piquette-Miller M., Allen C. (2008). Influence of molecular organization and interactions on drug release for an injectable polymer-lipid blend. Int. J. Pharm..

[172] Tan H., Peng Z., Li Q., Xu X., Guo S., Tang T. (2012). The use of quaternised chitosan-loaded PMMA to inhibit biofilm formation and downregulate the virulence-associated gene expression of antibiotic-resistant staphylococcus. Biomaterials.

[173] Huang T.W., Ho Y.C., Tsai T.N., Tseng C.L., Lin C., Mi F.L. (2020). Enhancement of the permeability and activities of epigallocatechin gallate by quaternary ammonium chitosan/fucoidan nanoparticles. Carbohydr. Polym..

[174] Fortage J., Tuyèras F., Peltier C., Dupeyre G., Calboréan A., Bedioui F., Ochsenbein P., Puntoriero F., Campagna S., Ciofini I. (2012). Tictoid expanded pyridiniums: Assessing structural, electrochemical, electronic, and photophysical features. J. Phys. Chem. A.

[175] Madaan P., Tiyagi V.K. (2008). Quaternary pyridinium salts: A review. J. Oleo Sci..

[176] Vogel A. (1989). Vogel-A Text-Book of Practical Organic Chemistry.

[177] Haldar J., Kondaiah P., Bhattacharya S. (2005). Synthesis and antibacterial properties of novel hydrolyzable cationic amphiphiles. Incorporation of multiple head groups leads to impressive antibacterial activity. J. Med. Chem..

[178] Ilangovan A., Venkatesan P., Sundararaman M., Kumar R.R. (2012). Synthesis, characterization and antimicrobial activity of 4-amino-1-alkyl pyridinium salts. Med. Chem. Res..

[179] Sowmiah S., Esperança J.M., Rebelo L.P., Afonso C.A. (2018). Pyridinium salts: From synthesis to reactivity and applications. Org. Chem. Front..

[180] Li R., Guo Z., Jiang P. (2010). Synthesis, characterization, and antifungal activity of novel quaternary chitosan derivatives. Carbohydr. Res..

[181] Guo Z., Chen R., Xing R., Liu S., Yu H., Wang P., Li C., Li P. (2006). Novel derivatives of chitosan and their antifungal activities in vitro. Carbohydr. Res..

[182] Tan W., Li Q., Gao Z., Qiu S., Dong F., Guo Z. (2017). Design, synthesis of novel starch derivative bearing 1,2,3-triazolium and pyridinium and evaluation of its antifungal activity. Carbohydr. Polym..

[183] Li Q., Zhang C., Tan W., Gu G., Guo Z. (2017). Novel amino-pyridine functionalized chitosan quaternary ammonium derivatives: Design, synthesis, and antioxidant activity. Molecules.

[184] Vetter A.C., Nikitin K., Gilheany D.G. (2018). Long sought synthesis of quaternary phosphonium salts from phosphine oxides: Inverse reactivity approach. Chem. Commun..

[185] Kenawy E.R., Kandil S. (2014). CHAPTER 3 Synthesis, Antimicrobial Activity and Applications of Polymers with Ammonium and Phosphonium Groups. Polymeric Materials with Antimicrobial Activity: From Synthesis to Applications.

[186] Guo A., Wang F., Lin W., Xu X., Tang T., Shen Y., Guo S. (2014). Evaluation of antibacterial activity of N-phosphonium chitosan as a novel polymeric antibacterial agent. Int. J. Biol. Macromol..

[187] Zhu D., Cheng H., Li J., Zhang W., Shen Y., Chen S., Ge Z., Chen S. (2016). Enhanced water-solubility and antibacterial activity of novel chitosan derivatives modified with quaternary phosphonium salt. Mater. Sci. Eng. C.

[188] Tan W., Li Q., Dong F., Chen Q., Guo Z. (2017). Preparation and characterization of novel cationic chitosan derivatives bearing quaternary ammonium and phosphonium salts and assessment of their antifungal properties. Molecules.

[189] Fan L., Yang J., Wu H., Hu Z., Yi J., Tong J., Zhu X. (2015). Preparation and characterization of quaternary ammonium chitosan hydrogel with significant antibacterial activity. Int. J. Biol. Macromol..

[190] Qi F., Cai Z.S., Zhu X.M., Shang S.B., Pei L.J. (2015). Synthesis, characterization, and performance of a novel polymeric cationic surfactant based on low molecular weight chitosan and 3-chloro-2-hydroxypropyl dimethyl dehydroabietyl ammonium chloride (CHPDMDHA). J. Surfactants Deterg..

[191] Holappa J., Hjálmarsdóttir M., Másson M., Rúnarsson Ö., Asplund T., Soininen P., Nevalainen T., Järvinen T. (2006). Antimicrobial activity of chitosan N-betainates. Carbohydr. Polym..

[192] Holappa J., Nevalainen T., Safin R., Soininen P., Asplund T., Luttikhedde T., Másson M., Järvinen T. (2006). Novel water-soluble quaternary piperazine derivatives of chitosan: Synthesis and characterization. Macromol. Biosci..

[193] Korjamo T., Holappa J., Taimisto S., Savolainen J., Järvinen T., Mönkkönen J. (2008). Effect of N-betainate and N-piperazine derivatives of chitosan on the paracellular transport of mannitol in Caco-2 cells. Eur. J. Pharm. Sci..

[194] Zambito Y., Uccello-Barretta G., Zaino C., Balzano F., Di Colo G. (2006). Novel transmucosal absorption enhancers obtained by aminoalkylation of chitosan. Eur. J. Pharm. Sci..

[195] Zambito Y., Zaino C., Burchielli S., Carelli V., Serafini M.F., Di Colo G. (2007). Novel quaternary ammonium chitosan derivatives for the promotion of intraocular drug absorption. J. Drug Deliv. Sci. Technol..

[196] Cao Z., Liu W., Xiong J., Qu N., Li H., Yao G. (2011). Synthesis and properties of N,N-dimethyl-O-quaternary ammonium chitosan. Adv. Mater. Res..

[197] Li Z., Yang F., Yang R. (2015). Synthesis and characterization of chitosan derivatives with dual-antibacterial functional groups. Int. J. Biol. Macromol..

[198] Pan H., Zhao T., Xu L., Shen Y., Wang L., Ding Y. (2020). Preparation of novel chitosan derivatives and applications in functional finishing of textiles. Int. J. Biol. Macromol..

[199] Cai J., Dang Q., Liu C., Wang T., Fan B., Yan J., Xu Y. (2015). Preparation, characterization and antibacterial activity of O-acetyl-chitosan-N-2-hydroxypropyl trimethyl ammonium chloride. Int. J. Biol. Macromol..

[200] Zhang A., Ding D., Ren J., Zhu X., Yao Y. (2014). Synthesis, characterization, and drug-release behavior of amphiphilic quaternary ammonium chitosan derivatives. J. Appl. Polym. Sci..

[201] Pedro R.D.O., Schmitt C.C., Neumann M.G. (2016). Syntheses and characterization of amphiphilic quaternary ammonium chitosan derivatives. Carbohydr. Polym..

[202] Han Y., Lin Q. (2012). Synthesis, characterization, and antibacterial activity of quaternized of N-aromatic chitosan derivatives. Appl. Mech. Mater..

[203] Badawy M.E., Rabea E.I. (2014). Synthesis and antifungal property of N-(aryl) and quaternary N-(aryl) chitosan derivatives against Botrytis cinerea. Cellulose.

[204] Sang W., Tang Z., He M.Y., Hua Y.P., Xu Q. (2015). Synthesis and preservative application of quaternized carboxymethyl chitosan containing guanidine groups. Int. J. Biol. Macromol..

[205] Rahimi M., Ahmadi R., Samadi Kafil H., Shafiei-Irannejad V. (2019). A novel bioactive quaternized chitosan and its silver-containing nanocomposites as a potent antimicrobial wound dressing: Structural and biological properties. Mater. Sci. Eng. C.

